# *Zfp57* inactivation illustrates the role of ICR methylation in imprinted gene expression during neural differentiation of mouse ESCs

**DOI:** 10.1038/s41598-021-93297-3

**Published:** 2021-07-05

**Authors:** Basilia Acurzio, Ankit Verma, Alessia Polito, Carlo Giaccari, Francesco Cecere, Salvatore Fioriniello, Floriana Della Ragione, Annalisa Fico, Flavia Cerrato, Claudia Angelini, Robert Feil, Andrea Riccio

**Affiliations:** 1 Department of Environmental Biological and Pharmaceutical Sciences and Technologies (DiSTABiF), Università degli Studi della Campania “Luigi Vanvitelli”, 81100 Caserta, Italy; 2 Institute of Genetics and Biophysics (IGB), Adriano Buzzati-Traverso”, Consiglio Nazionale delle Ricerche (CNR), 80131 Naples, Italy; 3 Department of Biology, Università degli Studi di Napoli “Federico II”, 80126 Napoli, Italy; 4 Institute for Applied Mathematics (IAC) “Mauro Picone”, Consiglio Nazionale delle Ricerche (CNR), 80131 Naples, Italy; 5 Institute of Molecular Genetics of Montpellier (IGMM), Centre National de Recherche Scientifique (CNRS), 34293 Montpellier, France; 6 University of Montpellier, 34090 Montpellier, France; 7 Present Address: Department of Neuroscience and Brain Technologies, Istituto Italiano di Tecnologia, 16163 Genova, Italy

**Keywords:** Genetics, Epigenetics, Imprinting

## Abstract

ZFP57 is required to maintain the germline-marked differential methylation at imprinting control regions (ICRs) in mouse embryonic stem cells (ESCs). Although DNA methylation has a key role in genomic imprinting, several imprinted genes are controlled by different mechanisms, and a comprehensive study of the relationship between DMR methylation and imprinted gene expression is lacking. To address the latter issue, we differentiated wild-type and *Zfp57*^-/-^ hybrid mouse ESCs into neural precursor cells (NPCs) and evaluated allelic expression of imprinted genes. In mutant NPCs, we observed a reduction of allelic bias of all the 32 genes that were imprinted in wild-type cells, demonstrating that ZFP57-dependent methylation is required for maintaining or acquiring imprinted gene expression during differentiation. Analysis of expression levels showed that imprinted genes expressed from the non-methylated chromosome were generally up-regulated, and those expressed from the methylated chromosome were down-regulated in mutant cells. However, expression levels of several imprinted genes acquiring biallelic expression were not affected, suggesting the existence of compensatory mechanisms that control their RNA level. Since neural differentiation was partially impaired in *Zfp57*-mutant cells, this study also indicates that imprinted genes and/or non-imprinted ZFP57-target genes are required for proper neurogenesis in cultured ESCs.

## Introduction

In mammals, a subset of genes referred to as imprinted genes are expressed only from the allele that is inherited from the mother or only from the allele that is inherited from the father^1^. Currently, over 150 genes have been reported to be imprinted in the mouse, many of which are also imprinted in humans^2–5^. Most of these genes have important roles in fetal growth, development and neural functions, and their dysregulation results in diseases^1,6^. The majority of imprinted genes are arranged in chromosomal clusters that are controlled by imprinting control regions (ICRs)^7^. ICRs overlap CpG-rich sequences that display differential DNA methylation (differentially methylated regions, DMRs) and differential histone modifications on their maternal and paternal alleles. DNA methylation is differentially established at ICRs in male and female germlines and maintained throughout development, despite the global demethylation and subsequent de novo methylation occurring in early embryogenesis^8^. Seminal studies in the mouse have demonstrated the critical role of DNA methylation in maintaining imprinted gene expression^9,10^. However, several imprinted loci appear not to be regulated by DNA methylation^11–14^. More recently, a non-canonical and H3K27me3-dependent form of imprinting has been demonstrated in the mouse^15,16^.

The KRAB zinc finger protein ZFP57 has a critical role in maintaining DNA methylation at ICRs in both humans and mice^17,18^. Homozygous mutations of *ZFP57* have been reported in human individuals affected by type 1-transient neonatal diabetes mellitus (TNDM1), who present multi-locus imprinting disturbances. In the mouse, maternal-zygotic deletion of *Zfp57* results in failure to maintain DNA methylation imprints, and causes mid-gestation embryonic lethality. ZFP57 recognizes the methylated hexameric motif "T/GGCCGC'' within ICRs and recruits the corepressor KRAB-A-interacting protein (KAP1) together with DNA methyltransferases and histone H3K9 methyltransferases to these sites^19–22^. In addition to ICRs, ZFP57 binds numerous non-imprinted genes and many transposable elements in mouse embryonic stem cells (ESCs). However, upon *Zfp57* deletion, most or all ICRs lose their allelic DNA methylation and H3K9me3, while non-imprinted target loci lose H3K9me3 but maintain most of their DNA methylation, and repetitive elements are not apparently affected^23,24^. Recently, the KRAB zinc finger protein ZNF445/ZFP445 was identified as a further important regulator of genomic imprinting^25^. However, its role seems to be more relevant in humans, while in mice *Zfp445* appears to be required for preserving methylation at only a subset of ICRs in the absence of *Zfp57*^25^.

Loss of *Zfp57* results in expression changes at only a few imprinted and non-imprinted genes in pluripotent ESCs^19,23^. Allele-specific gene expression analysis in hybrid *Zfp57*^-/-^ ESCs demonstrated that loss of *Zfp57* causes derepression of the silenced allele of these imprinted loci^24^. However, despite the fact that most ICRs displayed loss of methylation, only a few imprinted genes had their allele-specific expression status deregulated in *Zfp57*^-/-^ ESCs. In fact, most imprinted genes have low RNA levels in pluripotent ESCs, but become activated in a lineage-specific manner during differentiation particularly during neurogenesis^26^. In this study, we investigated the effect of *Zfp57* inactivation upon differentiation of ESCs into neural precursor cells (NPCs). The results obtained highlight the complex relationship between ICR methylation maintained by ZFP57 and imprinted gene expression during neural differentiation.

## Results

### Deregulation of ZFP57 target genes in *Zfp57*^-/-^ ES cell-derived neural precursor cells

**Figure 1:**
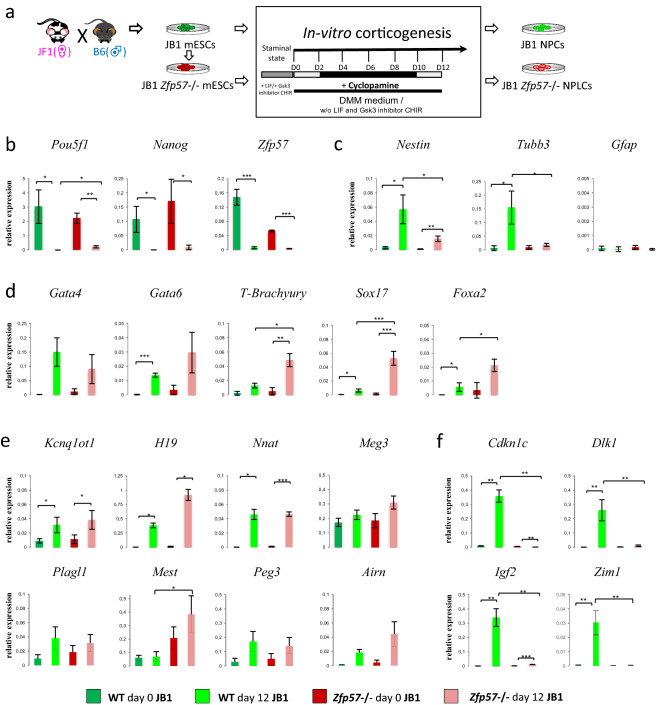
Differentiation protocol and marker expression analysis of wild-type and *Zfp57*^-/-^ hybrid cells. (**a**) Schematic of neural differentiation. Wild-type and *Zfp57*^-/-^ JB1 ESCs were cultured in DDM medium for 12 days and 1 μM cyclopamine was added from day 2 to day 10 to induce dorsalization of progenitor cells. At day 12 neural progenitor cells were harvested. (**b–d**) Expression analysis of pluripotency **(b)**, neural **(c)** and endoderm-mesoderm **(c)** differentiation markers and imprinted genes **(e,f)** in wild-type and *Zfp57*^-/-^ JB1 ESCs (day 0) and NPCs (day 12) assayed by quantitative RT-qPCR. The histograms show the average gene expression levels of three independent experiments, after normalization against the level of *β-actin*. Error bars represent the SD. Note that *Zfp57* expression was detected with the use of primers external to the deletion introduced during the gene knockout.

It was previously shown that in vitro corticogenesis can recapitulate the imprinting identity of perinatal cortex, and yields the correct parent-of-origin-dependent expression and DNA methylation at imprinted gene loci^26^. We reasoned, therefore, that this system could be a good model to test the consequences of *Zfp57* inactivation on genomic imprinting. Neural differentiation was induced in pluripotent ESCs, following an established protocol with addition of the sonic hedgehog inhibitor cyclopamine that generates cortical-identity neural progenitors^27^ (Fig. 1a). For this study, we used ESCs that were hybrid between the genetically divergent M. m. molossinus strain JF1 and C57BL6/J, so that at almost all imprinted genes the parental alleles could be told apart.

Wild-type (JB1) ESCs and the previously obtained JB1-derived *Zfp57*^-/-^ ESCs^24^ were subjected to the differentiation protocol, and the expression of pluripotency and neural markers was investigated by quantitative RT-PCR and immunofluorescence (IF, Fig. 1b,c, Supplementary Fig. 1). Differentiation was stopped at day 12 to obtain neural precursor cells (NPCs), in which it has been reported that the expression of many imprinted genes is activated^26^. The experiment was repeated several times, and down-regulation of the pluripotency markers *Nanog* and *Pou5f1* was always observed in both wild-type and *Zfp57*^-/-^ cells (Fig. 1b, Supplementary Fig. 1). In addition, the NPC-specific marker *Nestin* was increased from day 0 to day 12 in both wild-type and *Zfp57*^-/-^ cells, although its expression was found lower in the mutant cells by quantitative RT-PCR (qRT-PCR, Fig. 1c). Furthermore, down-regulation of the pluripotency marker Pou5f1 in all the cells and activation of Nestin in most of them was demonstrated at day 12 in the *Zfp57*-mutant and wild-type strain by IF (Supplementary Fig. 1). Consistent with cell differentiation of both cell lines, there was also down-regulation of *Zfp57*^28^*,* which was detected by qRT-PCR with the use of primers external to the deletion introduced during the knockout (Fig. 1b). Although these results demonstrate that after the 12 day-cyclopamine treatment the *Zfp57*-mutant cells lost their pluripotency markers and acquired a NPC-specific marker, the more terminal marker *Tubb3* was found activated only in the wild-type strain (Fig. 1c). Consistent with the differentiation protocol used (day 12)^26^, the glia-specific marker *Gfap* was not activated in either of the cell lines. Finally, some meso-endoderm-specific markers were activated with some differences in wild-type and mutant strains, suggesting that some of the cells also differentiated toward non-neural cell fates (Fig. 1d). Concerning the imprinted genes, significant activation of *Kcnq1ot1, H19* and *Nnat* was observed between day 0 and day 12 in both wild-type and mutant cells (Fig. 1e). A similar trend was observed for *Meg3*, *Plagl1*, *Mest*, *Peg3* and *Airn*, although the changes of the individual genes did not reach statistical significance. Conversely, *Cdkn1c*, *Dlk1, Igf2* and *Zim1* were activated upon differentiation only in the wild-type cells (Fig. 1f). A further *Zfp57* knockout clone, which was previously obtained in an inbred 129 ESC strain (E14^24^), was investigated by IF and qRT-PCR after the 12 day-cyclopamine treatment. This cell strain demonstrated Pou5f1 down-regulation, Nestin activation and deregulation of imprinted gene expression similar to the *Zfp57*^-/-^ JB1 cells (Supplementary Fig. 2). In summary, these results indicate that the ESCs lacking *Zfp57* are able to exit from pluripotency and partially differentiate into NPCs.

**Figure 2:**
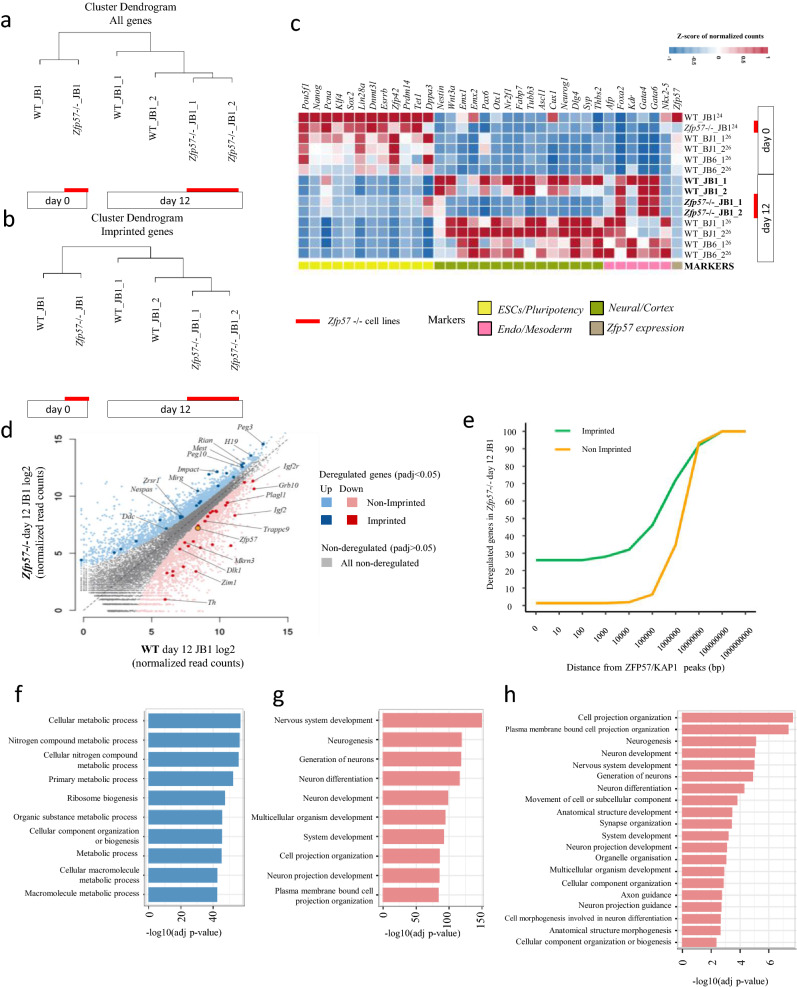
Whole-genome expression analysis of wild-type and *Zfp57*^-/-^ hybrid day-12 cells. (**a,b**) Cluster analysis of RNAseq data focusing on all genes (**a**) or imprinted genes (**b**) of day 0 and day 12-wild-type and *Zfp57*^-/-^ JB1 cells. *Zfp57*^-/-^ cells are indicated by a red line. (**c**) Heatmap representing lineage-specific marker expression in wild-type and *Zfp57*^-/-^ JB1 cells derived from RNAseq analysis. The public datasets (references indicated) were retrieved from the GEO database (Supplementary Table 1). The new datasets are indicated with bold characters. The heatmap was created using R package "pheatmap version 1.0.12" (https://CRAN.R-project.org/package=pheatmap). (**d**) Scatter plot showing deregulated genes in *Zfp57*^−/−^ day 12-JB1 versus wild-type day 12-JB1 cells. Deregulated genes are indicated by red dots if downregulated (non-imprinted genes by light-red; imprinted genes by dark-red) and by blue dots if upregulated (non-imprinted genes by light-blue; imprinted genes by dark-blue). (**e**) Cumulative distribution of the distances (bp) of deregulated genes (imprinted genes: green; non-imprinted genes: orange) in *Zfp57*^-/-^ day 12-JB1 cells from the ZFP57 target sites determined in wild-type day 0-JB1 cells^24^. The difference between the two curves is statistically significant according to the two-sample Kolmogorov–Smirnov test (p-value < 1e-10). (**f,g**) Gene ontology analysis of differentially expressed genes in *Zfp57*^−/−^ day 12-JB1 cells (f, upregulated; g, downregulated). Bars represent GO:BP terms sorted by high to low -log10 (adjusted p-value). (**h**) Gene ontology analysis of down-regulated genes located < 100 Kb from ZFP57-KAP1 binding sites.

Two biological replicates of day 12-differentiated wild-type and *Zfp57*^-/-^ JB1 cells were analyzed by RNA-seq. The results obtained were compared with the datasets from day 0-pluripotent wild-type and day 0-pluripotent *Zfp57*^-/-^ JB1 cells (one replicate each) previously obtained in our laboratory^24^, as well as with the datasets of day 0-pluripotent and day 12-differentiated wild-type JB1 and BJ1 cells (two replicates each) previously reported by Bouschet and collaborators^26^ (Fig. 2). Hierarchical clustering demonstrated that pluripotent and differentiated cells were separated, while wild-type and *Zfp57*-mutant cells grouped together, when either all genes or only imprinted genes were considered, indicating that gene expression of the wild-type and mutant strains can be compared (Fig. 2a,b). The analysis of the pluripotency and differentiation markers by RNA-seq confirmed the results obtained by RT-PCR and IF (Fig. 2c). In particular, while ESC markers were generally down-regulated in both wild-type and mutant day 12-JB1 cells, *Nestin* but no other neural markers were activated in the *Zfp57*^-/-^ cells at a level similar to that of their control cells, or to previously reported hybrid NPCs^26^. Nevertheless, activation of the endo-mesoderm markers *Foxa2*, *Gata4* and *Gata6* was similar in wild-type and mutant strains.

By using the DESeq2 software^29^, we identified 4912 differentially expressed genes in the *Zfp57*^-/-^ day 12-JB1 cells with respect to the day 12-JB1 wild-type cells. These included both up-regulated and down-regulated genes in similar proportions (Fig. 2d, Supplementary Table 2). 50 of these genes were reported to be imprinted^3, 4^, indicating that the differentially expressed genes were enriched for imprinted genes (p-value < 1e−04).

We previously showed that loss of *Zfp57* causes profound changes in DNA methylation and histone marks at its target sites in pluripotent ESCs^24^. To determine if the inheritance of these epigenetic modifications may have directly affected gene expression in NPCs, we calculated the distance of deregulated genes from previously mapped ZFP57 binding sites in ESCs^24^. We found that deregulated imprinted genes were more frequently within 1000 kb from ZFP57 targets than deregulated non-imprinted genes (Fig. 2e). While imprinted gene expression was mostly altered by defective ZFP57 binding to ICRs at the imprinted loci themselves, many deregulated non-imprinted genes were located far from ZFP57 sites and were likely indirectly affected.

We then examined the identity and functional classification of the deregulated genes by gene ontology analysis. While up-regulated genes were mostly linked to intracellular functions and metabolic processes, down-regulated genes were prevalently involved in neurogenesis and neuron development pathways (Fig. 2f,g). Down-regulated genes involved in neuronal and developmental functions included 6 imprinted genes and 68 non-imprinted genes that were located within 100 kb from the ZFP57 binding sites (Fig. 2h, Table 1 and Supplementary Table 3). Significantly, 24 of these non-imprinted genes were overlapping ZFP57 binding sites. This suggests that in addition to disrupting the expression of important imprinted genes, loss of ZFP57 binding may directly alter the expression of a group of non-imprinted genes that controls neuronal differentiation of ESCs in culture.

**Table 1:** *Zfp57*^-/-^ day 12-JB1 cells down-regulated genes located < 100 kb from ZFP57/KAP1 binding sites and involved in neuronal and developmental functions.

Gene^a^	Log2 fold change (*Zfp57*^-/-^ vs WT)	Distance from ZFP57 peak (bp)	Functional category^b^
*Ptpn5*	−5.650198389	24,245	1,2,3,4,5,6,8,9,10,14
*Hes5*	−5.178415842	9979	1,2,3,4,5,6,8,9,10,14,15,19
*Otx1*	−5.163770188	3925	3,6,8,10,14,15,19
*Pthlh*	−3.996723886	77,959	6,8,10,14
*Dner*	−3.840008981	0	1,3,6,8,10,14,15
*Trim67*	−3.77993524	9591	1,2,3,4,5,6,8,9,10,14
*Grem2*	−3.332414676	0	6,8,10,14
*Lama2*	−3.187941857	44,022	1,2,3,4,5,6,8,9,10,11,12,13,14,16,17,18
*Sobp*	−3.001779515	23,142	6,8,10,14
*Megf11*	−2.998280188	0	6,8,10,14
*Tubb3*	−2.961888079	4595	1,2,3,4,5,6,8,9,10,11,12,13,14,16,17,18
*Stx1b*	−2.953214561	43,156	1,2,3,4,5,6,8,9,10,14
*Unc5a*	−2.861952413	7113	1,2,3,4,5,6,8,9,10,11,12,13,14,16,17,18
*Iqsec3*	−2.855605248	45,489	7
***Igf2***	−2.800585832	68,640	6,8,10,14
*Sparcl1*	−2.622871614	16,805	6,7,14
*Cacna2d2*	−2.593159777	60,968	6,7,14
*Mapk8ip2*	−2.581849823	78,444	1,2,3,4,5,6,8,9,10,13,14,17
*Fgfr2*	−2.563740007	0	6,8,10,14
*Rfx3*	−2.525629394	66,048	6,8,10,14
*Kcnk2*	−2.496481463	65,056	6,8,10,14
*Adgrv1*	−2.447653122	30,325	1,2,3,4,5,6,8,9,10,14
*Slit1*	−2.447307746	0	1,2,3,4,5,6,7,8,9,10,11,12,13,14,15,16,17,18,19
*Nefl*	−2.377539166	32,318	1,2,3,4,5,6,7,8,9,10,13,14,15,16,17,18,19
*Ank3*	−2.289887609	0	1,2,3,4,5,6,7,8,9,10,11,12,13,14,16,17,18
*Prkcb*	−2.05207033	0	6,8,10,14
*Tubb2a*	−1.924739676	88,398	1,3,4,6,8,10,14
*Aldh1a2*	−1.878207424	0	1,3,4,5,6,8,10,14,15,19
*Tubb2b*	−1.835573303	35,939	1,2,3,4,5,6,8,9,10,11,12,13,14,16,17,18
*Lgi2*	−1.728979281	17,027	3,6,7,8,10,14
*Lama4*	−1.717209218	30,669	6,8,10,14
*Akt3*	−1.607596025	39,078	3,6,8,10,14,15,19
*Fzd9*	−1.601739355	98,514	1,3,4,6,7,8,10,14
*Tenm4*	−1.574916915	0	1,2,3,4,5,6,8,10,14,15
*Ttbk2*	−1.438103277	96,955	3,6,8,10,14,15,19
*Efna5*	−1.37690116	27,126	1,2,3,4,5,6,7,8,9,10,11,12,13,14,15,16,17,18,19
*Ankrd6*	−1.349451504	0	6,8,10,14
*Cadm1*	−1.323861372	0	3,6,7,8,10,14
*Tub*	−1.323006164	0	6,8,10,14
*Id1*	−1.309638653	49,256	1,2,3,4,5,6,8,9,10,13,14,15,17,19
*Hnrnph3*	−1.273951816	87,066	6,14
*Bbs1*	−1.237741962	50,963	1,2,3,4,5,6,8,9,10,13,14,15,19
*Fgd4*	−1.19828348	43,220	6,14
*Gnaq*	−1.17374567	0	1,2,3,4,5,6,8,10,14,15,19
*Prkca*	−1.156879418	0	6,8,10,14
*Gpr137*	−1.156856043	56,855	6,8,10,14
***Plagl1***	−1.127612179	0	6,8,10,14
*Sdccag8*	−1.110806796	0	1,3,4,6,8,10,14
***Igf2r***	−1.108075081	0	6,8,10,14
***Trappc9***	−1.106009642	0	1,3,4,5,6,8,10,14,15,19
*Kif3a*	−1.083661795	0	1,2,3,4,5,6,8,9,10,11,12,13,14,15,16,17,18,19
*Meis3*	−1.054682659	78,401	3,6,8,10,14,15,19
*Apbb1*	−1.054136643	37,175	1,2,3,4,5,6,7,8,9,10,11,12,13,14,16,17,18
*Sema3f.*	−1.050995148	90,917	1,2,3,4,5,6,7,8,9,10,11,12,13,14,15,16,17,18
*Shank3*	−1.033118722	0	1,2,3,4,5,6,7,8,9,10,13,14,15,17,19
***H13***	−1.011379813	0	6,10,14
*Limk1*	−1.003120469	97,408	1,2,3,4,5,6,8,9,10,13,14,16,17,18
*Palm*	−0.9827988076	53,726	3,6,7,8,10,14
*Naglu*	−0.9714420126	95,071	1,2,3,4,5,6,8,10,14,15,19
*Arhgef7*	−0.9214359692	0	1,3,6,7,8,10,14
*Fam171a1*	−0.8829257793	0	6,14
*Arl3*	−0.865781179	15,058	1,2,3,4,5,6,8,10,14
*Usf3*	−0.8508211011	17,582	6,8,10,14
*Ank*	−0.8178055036	0	6,8,10,14
*Kdm1b*	−0.8026958823	32,233	6,10,14
*Kat6a*	−0.7545602154	0	6,8,10,14
***Inpp5f***	−0.7516626695	0	1,2,3,4,5,6,8,9,10,14,16
*Exoc5*	−0.7432131534	60,611	6,14
*Phc2*	−0.6955736462	68,910	6,10,14
*Pdzrn3*	−0.6821083965	0	7
*Alg10b*	−0.6296632078	0	1,2,3,4,5,6,8,10,14
*Gpi1*	−0.6184329932	22,645	6,10,14
*Trio*	−0.5863515137	0	1,2,3,4,5,6,8,9,10,11,12,13,14,15,16,17,18
*Ptprg*	−0.5802367554	1086	1,2,3,4,5,6,8,9,10,14,15,19

^a^Imprinted genes are in bold. ^2^GO Category are as follows: 1. GO:0022008(neurogenesis), 2. GO:0048666(neuron development), 3. GO:0007399(nervous system development), 4. GO:0048699(generation of neurons), 5. GO:0030182(neuron differentiation), 6. GO:0048856(anatomical structure development), 7. GO:0050808(synapse organization), 8. GO:0048731(system development), 9. GO:0031175(neuron projection development), 10. GO:0007275(multicellular organism development), 11. GO:0007411(axon guidance), 12. GO:0097485(neuron projection guidance), 13. GO:0048667(cell morphogenesis involved in neuron differentiation), 14. GO:0032502(developmental process), 15. GO:0007417(central nervous system development), 16. GO:0061564(axon development), 17. GO:0048812(neuron projection morphogenesis), 18. GO:0007409(axonogenesis), 19. GO:0007420(brain development).

Overall, these results demonstrate that when exposed to the differentiation conditions, *Zfp57*^-/-^ ESCs exit from the pluripotency state and up-regulate some differentiation markers, but fail to fully activate neural markers compared to the wild-type cells. Consistent with these findings, down-regulated genes were prevalently involved in neurogenesis and neuron development pathways. However, although expression differences between the *Zfp57*^-/-^ and wild-type cells were extensive and affected both imprinted and non-imprinted genes, both gene and protein expression analyses demonstrated that the differentiated mutant cells were significantly different from pluripotent ESCs, likely undergoing a partial differentiation towards the neural fate. Therefore, we went on to analyze the consequence of ZFP57 loss on the allele-specificity of imprinted gene expression in these neural precursor-like cells (NPLCs).

### Massive deregulation of allele-specific expression of imprinted genes in *Zfp57*^-/-^ cells after differentiation

We previously demonstrated that pluripotent ESCs lose ICR methylation upon *Zfp57* inactivation^24^. Now, we investigated ICR methylation levels in wild-type and *Zfp57*^-/-^ day 12 cells, to see if any change had occurred during differentiation of pluripotent ESCs. Methylation of 10 ICRs (7 maternally methylated and 3 paternally methylated loci) was determined by several methods. In particular, by bisulfite treatment, cloning and sequencing we demonstrated that differential methylation of the paternal and maternal alleles was maintained in the wild-type JB1 cells at day 12 but was not acquired in the day 12-*Zfp57*^-/-^ JB1 cells at the *Plagl1*, *Peg13* and *Igf2r* ICRs (Supplementary Fig. 3a). In addition, maintenance of the expected 50% methylation level in the wild-type day 0-JB1 and E14 cells and low level of methylation in their cognate *Zfp57*-mutant cells after differentiation was demonstrated by bisulfite conversion followed by pyrosequencing or direct Sanger sequencing at the *Snrpn*, *Peg3*, *Kcnq1ot1*, *Dlk1*-*Meg3*, *Mest* and *H19* ICRs (Supplementary Fig. 3b–d). Thus, consistent with previous reports, imprinted ICR methylation was maintained in the wild-type NPCs^26^. Conversely, the ICR methylation levels remained very low in the *Zfp57*^-/-^ NPLCs, demonstrating that the loss of DNA methylation induced by ZFP57 depletion in pluripotent ESCs was maintained during in vitro corticogenesis.

**Figure 3:**
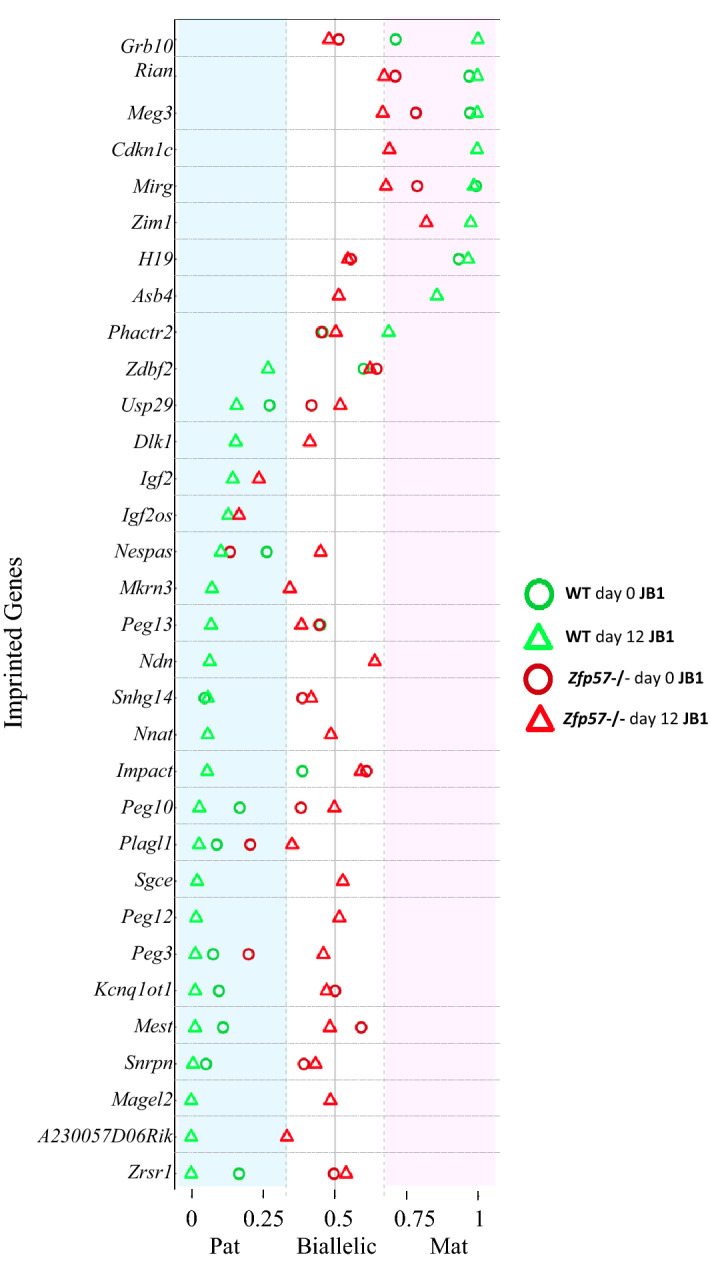
Allele-specific expression of imprinted genes in the wild-type and *Zfp57*^-/-^ day 0 and day 12-JB1 cells. Dot plot representing the allelic expression of imprinted genes in wild-type and *Zfp57*^-/-^ day 0- (green and red circles, respectively) and wild-type and *Zfp57*^-/-^ day 12- (green and red triangles, respectively) JB1 cells. The blue-shaded area corresponds to paternal (B6)-specific expression (allelic ratio interval: 0–0.33), the pink-shaded area corresponds to maternal (JF1)-specific expression (allelic ratio interval: 0.67–1), the white area corresponds to biallelically expressed genes (allelic ratio > 0.33 or < 0.67). The allelic ratio is the average of two biological duplicates (for details, see Supplementary Table 5). The data of wild-type and *Zfp57*^-/-^ ESCs are derived from a previous study^24^.

To determine the consequences of *Zfp57* loss on imprinted gene expression, we analysed the RNA-Seq data obtained from NPCs in an allele-specific manner. The mouse ESC lines used for neural differentiation derived from inter-sub-species (JF1 × C57BL6/J)F1 hybrid blastocysts. The genetic divergence between the C57BL6/6 and JF1 inbred mouse strains (> 4 SNPs/kb) allowed us to distinguish the parental origin of most imprinted genes. Only for a few protein-coding genes, including *Gatm*, *H13*, *Mcts2*, *Zim2*, and for the *Snord115-6* cluster and many miRNAs, allele-specific expression could not be measured because of absence of suitable SNPs. After removal of genes with low-counts and genes located on the X chromosome (the JB1 ESCs are male), allele-specific expression was determined by calculating the mat/(mat + pat) allelic ratio for each gene in the RNA-seq datasets. The genes with an average allelic ratio within the interval 0–0.33 were arbitrarily considered paternally expressed, and the genes with an average allelic ratio in the interval 0.67–1 were considered maternally expressed. In our previous study on pluripotent ESCs, we identified only a few genes with imprinted expression^24^. We have now re-evaluated the RNAseq dataset of pluripotent ESCs reported in ref.^24^ together with the new NPC dataset by using SNPsplit, an algorithm which efficiently splits the reads between genomes with known SNP genotypes (Supplementary Table 4)^30^. By following this approach, 15 imprinted genes demonstrated significant parent-of-origin-dependent allelic expression in the wild-type day 0-JB1 cells and 32 in the day 12-JB1 cells (Fig. 3 and Supplementary Table 5). Among the NPC imprinted genes, 9 were predominantly expressed from the maternal allele, and 23 from the paternal allele, consistent with what was previously demonstrated in mouse tissues^4^. The genes that were imprinted in the NPCs included genes that were either not imprinted in ESCs (e.g. *Zdbf2, Phactr2*, *Peg13* and *Impact*) or whose expression in ESCs was too weak to assess allele-specificity (Supplementary Table 5). With a few exceptions, these results are consistent with those described in the study of Bouschet and collaborators^26^.

We next analyzed the allele-specific expression of the imprinted genes in the *Zfp57*-mutant JB1 cells at day 0 and day 12 of cyclopamine treatment. We found that all the genes (but *Nespas* in ESCs) that were imprinted in wild-type cells, reduced their allelic expression bias in the *Zfp57*^-/-^ cells (Fig. 3, Supplementary Fig. 4 and Supplementary Table 5). However, the shift toward equivalent expression of the maternal and paternal alleles was more dramatic in the mutant cells at day 12 than at day 0. Notably, several genes (e.g. *Nespas*, *Peg3*, *Plagl1*, *Meg3*, *Rian* and *Mirg*) maintained a significant allelic bias in the mutant ESCs. The *Zdbf2*, *Peg13* and *Impact* genes, which were not imprinted in wild-type and mutant ESCs, acquired imprinting in the wild-type NPCs, but not in the mutant NPLCs.

### Loss of allelic bias is associated with different expression patterns of imprinted genes in *Zfp57*^-/-^ NPCs

To better understand the consequence of ZFP57 loss on genomic imprinting in neural differentiation, we integrated the results of gene expression levels with the allelic ratios determined in the *Zfp57*^-/-^ day 12-JB1 cells. An overall complex relationship between imprinting status and gene expression level was observed (Table 2). For several genes, the lack of biased allelic expression corresponded to global up-regulation of their RNA level, for others loss of imprinting was associated with down-regulation of their RNA levels. For the remaining ones, the loss of allele-specificity was not accompanied by a corresponding change in global expression levels.

**Table 2:** Integration of global gene expression with allele-specific expression of imprinted genes in *Zfp57*^-/-^ day 12-JB1 cells.

Domain	Chromosome	ICR methylation	Gene	Allelic expression wild-type NPCs	Allelic expression *Zfp57*^-/-^ NPCs	Global expression change
*Gpr1*	chr1	Maternal	*Zdbf2*	Paternal	Biallelic	Up-regulated
			*Adam23*	Biallelic	Biallelic	Up-regulated
*Nnat*	chr2	Maternal	*Nnat*	Paternal	Biallelic	Unchanged
			*Blcap*	Biallelic	Biallelic	Down-regulated
*Gnas*	chr2	Maternal	*Nespas*	Paternal	Biallelic	Up-regulated
*Peg10*	chr6	Maternal	*Sgce*	Paternal	Biallelic	Unchanged
			*Peg10*	Paternal	Biallelic	Up-regulated
			*Asb4*	Maternal	Biallelic	Unchanged
*Mest*	chr6	Maternal	*Mest*	Paternal	Biallelic	Up-regulated
*Peg3*	chr7	Maternal	*Zim1*	Maternal	All. bias reduced	Down-regulated
			*Peg3*	Paternal	Biallelic	Up-regulated
			*Usp29*	Paternal	Biallelic	Unchanged
*Snrpn*	chr7	Maternal	*Snhg14*	Paternal	Biallelic	Unchanged
			*Snrpn*	Paternal	Biallelic	Up-regulated
			*A230057D06Rik*	Paternal	Biallelic	Down-regulated
			*Ndn*	Paternal	Biallelic	Unchanged
			*Magel2*	Paternal	Biallelic	Unchanged
			*Mkrn3*	Paternal	Biallelic	Down-regulated
			*Peg12*	Paternal	Biallelic	Down-regulated
*H19*	chr7	Paternal	*H19*	Maternal	Biallelic	Up-regulated
			*Igf2*	Paternal	All. bias reduced	Down-regulated
			*Igf2os*	Paternal	Uncertain	Down-regulated
*Kcnq1ot1*	chr7	Maternal	*Th*	Maternal	Biallelic	Down-regulated
			*Kcnq1ot1*	Paternal	Biallelic	Unchanged
			*Cdkn1c*	Maternal	Biallelic	Down-regulated
*Plagl1*	chr10	Maternal	*Plagl1*	Paternal	Biallelic	Down-regulated
			*Phactr2*	Maternal	Biallelic	Unchanged
*Grb10*	chr11	Maternal	*Grb10*	Maternal	Biallelic	Down-regulated
			*Ddc*	Paternal^a^	All. bias inverted	Up-regulated
*Commd1*	chr11	Maternal	*Zrsr1*	Paternal	Biallelic	Up-regulated
*Meg3*	chr12	Paternal	*Dlk1*	Paternal	Biallelic	Down-regulated
			*Meg3*	Maternal	Biallelic	Unchanged
			*Rian*	Maternal	Biallelic	Up-regulated
			*Mirg*	Maternal	Biallelic	Up-regulated
*Trappc9*	chr15	Maternal	*Kcnk9*	Pref. maternal^a^	Biallelic	Down-regulated
			*Peg13*	Paternal	Biallelic	Unchanged
			*Trappc9*	Biallelic	Biallelic	Down-regulated
*Igf2r*	chr17	Maternal	*Igf2r*	Pref. maternal^a^	All. bias inverted	Down-regulated
			*Airn*	Paternal^a^	Biallelic	Up-regulated
*Impact*	chr18	Maternal	*Impact*	Paternal	Biallelic	Up-regulated

^a^Not statistically significant.

**Figure 4:**
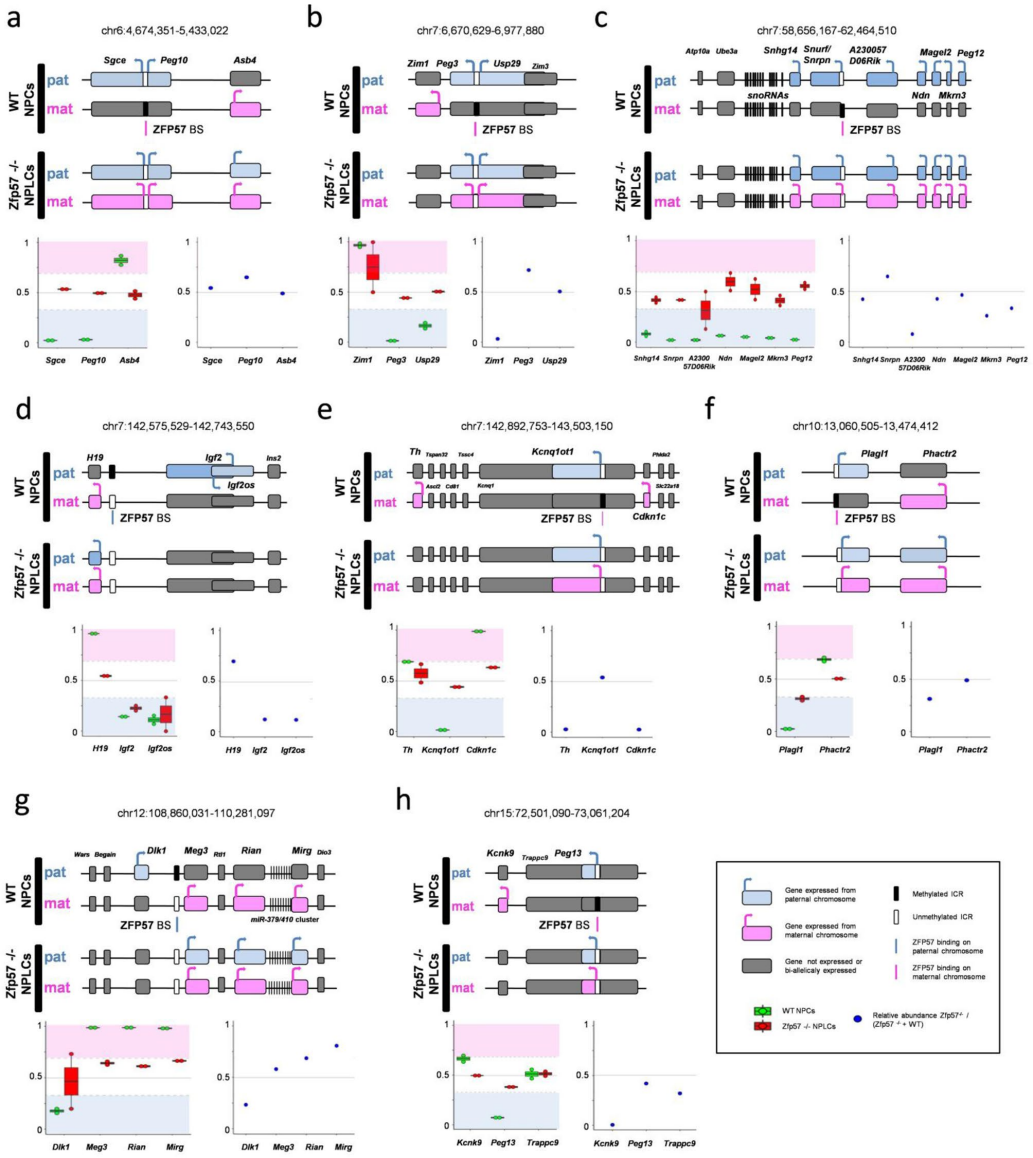
Coordinated change of allelic and levels of expression at imprinted gene clusters in the *Zfp57*^-/-^ day 12 cells. Panels (**a–h)** represent 8 different imprinted gene domains. A schematic of the gene cluster with indication of the imprinting status in wild-type and *Zfp57*^-/-^ day 12-JB1 cells, and location of the ZFP57 binding site (ZFP57 BS) is shown at the top of each panel. In each panel, the box-plot in the lower left shows the allelic expression ratio of the genes of the domain in the wild type (green) and *Zfp57*^-/-^ (red) day 12-JB1 cells. The dot-plot in the lower right shows the bulk gene expression changes detected in *Zfp57*^-/-^ day 12-JB1 cells (*Zfp57*^*-/-*^ day 12 / (wild-type day 12 + *Zfp57*^-/-^ day 12)). All values correspond to the average of two biological duplicates (for details, see Supplementary Tables 2, 4 and 5).

The effect of ZFP57 deficiency on imprinted gene expression is better understood if each affected imprinted domain is described separately (Fig. 4 and Supplementary Fig. 5). On chromosome 1, the imprinted domain including the *DBF-type zinc finger-containing protein 2* gene (*Zdbf2*) is controlled by a maternal germline DMR that is located within the *Gpr1* gene^31^. This DMR regulates the paternal-specific expression of long isoforms of *Zdbf2* (*Liz*), which in turn activates the canonical *Zdbf2* promoter on the paternal chromosome during embryonic development^31^. ZFP57 binds the *Gpr1* DMR on its methylated maternal allele, which loses its methylation in *Zfp57*^-/-^ ESCs^24^. Paternal-specific expression of *Zdbf2* was demonstrated in the wild-type day 12-cells, while this gene was activated twofold and imprinted expression was not acquired in the *Zfp57*^-/-^ cells cultured in the same conditions (Supplementary Fig. 5a). Another gene, *Adam23*, reported to be preferentially expressed from its paternal allele in mouse brain^32^, is located 100 kb from *Zdbf2,* in the same transcription orientation. We found *Adam23* biallelically expressed in both the wild-type and *Zfp57*^-/-^ cells, but observed a twofold activation of the global expression of this gene in the mutant cells, suggesting a coordinated regulation of this imprinted domain (Supplementary Fig. 5a).

In the *Blcap/Nnat* domain, a germline DMR overlaps the *Nnat* promoter that in turn is located in a *Blcap* intron^33^. ZFP57 interacts with this region in mouse ESCs, confirming its identity as ICR. We found paternal-specific expression of *Nnat* in the wild-type but imprinting was not acquired by this gene in the *Zfp57*-mutant day 12-cells, demonstrating a direct role of ZFP57-dependent methylation in the imprinting control of this locus (Supplementary Fig. 5b). However, differently from *Zdbf2*, the global mRNA expression level of *Nnat* was unchanged in the *Zfp57*-mutant cells. *Blcap* appears slightly maternally expressed in the wild-type day 12-cells, but its allelic ratio does not fall in the fixed intervals of imprinted expression, possibly because of the presence of non-imprinted isoforms^34^. However, the almost twofold down-regulation of this gene in the *Zfp57-*mutant day 12-cells confirms the hypothesis that *Blcap* imprinting is co-regulated with *Nnat* by transcriptional interference (Supplementary Fig. 5b).

Imprinting of the *Gnas* domain is controlled by the paternally expressed lncRNA *Nespas*^35^. *Nespas* downregulates its sense counterpart *Nesp,* which in turn controls the reciprocal gamete of origin-specific expression of *Gnas* and *GnasXL*. The *Nespas* promoter overlaps a germline DMR that is maternally methylated and bound by ZFP57 in mouse ESCs^24^. We found *Nespas* expressed from its paternal allele in the wild-type but activated on both parental alleles with an overall twofold increase of its mRNA level in the *Zfp57-*mutant day 12-cells, consistent with ZFP57-dependent imprinting regulation (Supplementary Fig. 5c). Unfortunately, *Nesp* expression was too low to determine its allelism in NPCs, and assess the regulatory role of ZFP57-dependent methylation on this gene.

A ZFP57-marked maternally methylated germline DMR overlaps the promoters of *Sgce* and *Peg10,* on proximal chromosome 6^36^. Consistent with *Zfp57*-dependent imprinting control of these two genes, we observed their paternal-specific expression in wild-type and bi-allelic expression in *Zfp57*-mutant day 12-cells (Fig. 4a). The change in global mRNA levels of these two genes, however, was not consistent: *Peg10* was up-regulated twofold, while *Sgce* displayed similar levels in wild-type and mutant cells. Another imprinted gene, *Asb4*, lies 700 kb distal from *Sgce/Peg10.* We found that *Asb4* was preferentially expressed from the maternal allele in the wild-type and lost its allelic bias in the mutant day 12-cells, although its global mRNA level was unchanged (Fig. 4a). Since the *Sgce/Peg10* DMR is the closest ZFP57 binding site in mouse ESCs^24^, this finding is consistent with the regulation of *Asb4* by this ICR. Of note, two further imprinted genes of this cluster that are highly expressed in neural cells, namely *Casd1* and *Ppp1r9a*^37^*,* were bi-allelically expressed in wild-type but down-regulated in the *Zfp57-*mutant day 12-cells. This suggests co-regulation of these genes with the rest of the cluster and is consistent with the incomplete neural differentiation of the *Zfp57*^*-/-*^ cells.

A further maternally methylated germline DMR is present on chromosome 6 and overlaps the promoter of *Peg1/Mest*^38^. Consistent with regulation by this ZFP57-bound DMR, we found that *Peg1/Mest* and its longer isoform *MestXl* were paternally expressed in the wild-type and bi-allelically expressed in the *Zfp57-*mutant day 12-cells (Supplementary Fig. 5d). We also observed an almost twofold up-regulation of *Peg1/Mest* in mutant cells. Although we did not detect any allelic bias for *Copg2*, this gene was down-regulated in *Zfp57*-mutant cells, consistent with the hypothesis that its expression is controlled by the overlapping anti-sense *MestXL*^38^. A further chromosome 6 gene, *Cntn3*, reported as imprinted in placenta but not in brain where it is highly expressed^39^, was bi-allelically expressed in the wild-type but strongly down-regulated in the *Zfp57*-mutant day 12-cells cells. This gene is not located close to an imprinted domain, but a ZFP57 binding site is located 1 Mbp from its transcription binding site^24^. Its repression may be caused by loss of ZFP57 binding at this site, although it could also be a consequence of the incomplete neural differentiation of the *Zfp57*-null cells.

On proximal chromosome 7, a ZFP57-bound DMR overlaps the promoters of the divergently transcribed *Peg3* and *Usp29* genes (Fig. 4b)^24^. Consistent with their imprinting being regulated by this DMR, *Peg3* and *Usp29* were both paternally expressed in the wild-type but bi-allelically expressed in the *Zfp57*-mutant day 12-cells. However, at the global level, *Peg3* was activated twofold while *Usp29* expression remained unchanged. Within the same domain, the maternally expressed *Zim1* also reduced its allelic bias, but its expression was strongly down-regulated in the mutant day 12-cells, consistent with the hypothesis that *Peg3* is a repressor of *Zim1*^40^.

In the central part of chromosome 7, another ZFP57-bound DMR overlaps the promoter of *Snurf/Snrpn*. This gene encodes large alternatively spliced transcripts, including the 460 kb lncRNA *Snhg14,* which were paternally expressed in the wild-type but were bi-allelic in the *Zfp57*-mutant day 12-cells (Fig. 4c). This cluster includes several other paternally expressed genes (*A230057D06Rik*, *Ndn*, *Magel2*, *Mkrn3* and *Peg12*) that all lost or reduced their allelic bias in *Zfp57*-mutant cells, indicating that they are coordinately controlled by methylation of the above mentioned DMR. Concerning their global RNA level, only *Snrpn* was up-regulated almost twofold, while the others were either unchanged or down-regulated.

On distal chromosome 7, two ZFP57-bound DMRs mark two adjacent imprinted domains. The more centromeric domain includes the *H19* and *Igf2* genes. These both displayed imprinted expression in the wild-type and lost or reduced, respectively, their allelic bias in the *Zfp57*-mutant day 12-cells (Fig. 4d). However, the paternally expressed *H19* was activated and *Igf2* repressed, consistent with the model of the paternally methylated insulator corresponding with the ZFP57-bound DMR^1,41^. Interestingly, the *Igf2* antisense transcript *Igf2os* was down-regulated similar to *Igf2*, indicating that it is controlled through a similar mechanism. The more distal ZFP57-bound DMR overlaps the promoter of the antisense noncoding gene *Kcnq1ot1*, which was found paternally expressed in the wild-type and bi-allelically expressed in the mutant day 12-cells, confirming its dependence from DMR methylation (Fig. 4e)^1^. The global level of the *Kcnq1ot1* RNA was unchanged. Consistent with the model predicting the regulation of this domain through the *cis*-repressive function of *Kcnq1ot1*, the closely located *Cdkn1c* gene was down-regulated and its maternal expression bias reduced. *Th* was also repressed but its allelic bias was borderline (adj. p-value = 0.0493). Another gene, *Ano1*, which lies 1 Mbp from the *Kcnq1ot1* DMR and was reported to be maternally expressed in placenta^42^, was found down-regulated in the *Zfp57*-mutant day 12-cells and therefore possibly coordinately regulated with the *Kcnq1ot1* domain, although no allelic expression bias was detected in the wild-type NPCs.

A further ZFP57-bound DMR overlaps with the *Plagl1* promoter on chromosome 10^24^. Consistent with regulation by this maternally methylated DMR, the *Plagl1* gene was found paternally expressed in the wild-type and bi-allelically expressed in the *Zfp57*-mutant day 12-cells (Fig. 4f). At the global level, the *Plagl1* mRNA was down-regulated in the mutant cells. The overlapping noncoding RNA *Hymai* is not currently mapped by UCSC, but according to the enrichment of the reads corresponding to its location (*Plagl1* intron 1), it appears to have the same behavior as *Plagl1*. The imprinting status of the close-by *Phactr2* gene is controversial^26,43^. However, we observed that *Phactr2* was preferentially expressed from the maternal allele in the wild-type cells, and its allelic bias was abolished in the mutant cells, suggesting that its imprinting is co-regulated with *Plagl1,* although its global expression level was similar in the wild-type and mutant cells.

Chromosome 11 harbours two maternally methylated ZFP57-bound DMRs^24^. The proximal one overlaps a CTCF binding site and an alternative promoter of the *Grb10* gene^44^. It has been proposed that DNA methylation interferes with the enhancer-blocking function of this DMR thereby allowing activation of the main *Grb10* promoter on the maternal chromosome. Consistent with this hypothesis, we found *Grb10* expressed from the maternal allele in the wild-type and from both parental alleles in the *Zfp57*-mutant day 12-cells (Supplementary Fig. 5f). However, loss of imprinting was accompanied by down-regulation of its global mRNA level. The *Ddc* gene is located downstream of *Grb10*. *Ddc* showed a paternal expression bias in the wild-type day 12-cells, which is consistent with previous reports^26^, but its bias was not strong enough to be classified as imprinted under our stringent criteria. However, its allelic expression was inverted and its global expression activated in the *Zfp57*^-/-^ day 12-cells, indicating that this gene may also be regulated by the *Grb10* methylation-sensitive insulator. The distal chromosome 11 DMR overlaps the promoter of the *Zrsr1* gene. *Zrsr1* was expressed from its paternal allele in the wild-type and up-regulated twofold on both parental alleles in the *Zfp57*-mutant day 12-cells, consistent with its regulation from its maternally methylated DMR (Supplementary Fig. 5e). We did not find any allelic expression bias of the overlapping *Commd1* gene in NPCs.

In the large cluster of imprinted genes on chromosome 12, ZFP57 binds a paternally methylated DMR that is located between the *Dlk1* and *Meg3* genes. We found *Dlk1* paternally expressed and *Meg3* maternally expressed in the wild-type but these genes were expressed from both parental alleles in the *Zfp57*-mutant day 12-cells, consistent with the model predicting imprinting dependent on the intergenic DMR methylation (Fig. 4g). In addition, *Dlk1* was down-regulated, consistent with the repressor function of the lncRNA *Meg3*^45^. A number of other genes (including *Mirg* and *Rian*) that are co-transcribed with *Meg3* within a polycistronic RNA drastically reduced their allelic bias (Supplementary Fig. 6a). Although the global RNA level of *Meg3* was only slightly changed, those of the small nucleolar RNA-containing gene *Rian* and the micro RNA-containing gene *Mirg* were up-regulated twofold and fourfold, respectively, in the mutant day 12-cells.

On chromosome 15, a ZFP57-bound DMR overlaps the promoter of the lncRNA gene *Peg13*^24^. This gene was paternally expressed in the wild-type and biallelically expressed in the *Zfp57*-mutant day 12-cells, consistent with being regulated by maternal methylation of this DMR, although its global expression was unchanged (Fig. 4h). The allelic bias of *Trappc9,* which overlaps *Peg13,* and that of *Kcnk9* located at its 3’ were not statistically significant in the wild-type but both of these genes were down-regulated in the *Zfp57*-mutant day 12-cells , consistent with their co-regulation with *Peg13.*

On chromosome 17, the promoter of the lncRNA *Airn* overlaps a maternally methylated and ZFP57-bound DMR^24^. Consistent with the model predicting transcriptional interference between *Airn* and *Igf2r*^46^, the former gene was activated and the latter repressed in the *Zfp57*-mutant day 12-cells (Supplementary Fig. 5 g). *Igf2r* appears slightly maternally expressed in the wild-type NPCs but its allelic ratio does not fall in the fixed intervals of imprinted expression, while *Airn* appears paternally expressed, but its counts are too low to perform a statistical test. However, the symmetrical changes of their allelic ratios observed in the *Zfp57*-mutant day 12-cells is consistent with their reciprocal imprinting being regulated by *Zfp57*-dependent DMR methylation.

Finally, on chromosome 18, ZFP57 marks the maternally methylated DMR overlapping the promoter of the *Impact* gene. The imprinting mechanism controlling *Impact* is poorly investigated. We found that this gene is paternally expressed in the wild-type and that the allelic bias is reduced in the mutant day 12-cells, with a global upregulation of about fourfold. These findings suggest that its imprinting is controlled by the ZFP57-bound DMR (Supplementary Fig. 5h).

In conclusion, all the imprinted genes detected in wild-type NPCs lost or reduced their allelic expression bias in the *Zfp57*^-/-^ NPLCs. However, those expressed from the non-methylated chromosome were generally up-regulated, and those expressed from the methylated chromosome were generally down-regulated. In addition, several genes (such as *Sgce*, *Asb4*, *Usp29, Snhg14, Ndn, Magel2, Kcnq1ot1*, *Phactr2, Meg3* and *Peg13*), whose expression was expected to be increased upon loss of ICR methylation, acquired biallelic expression but overall were not significantly activated.

## Discussion

Differential DNA methylation of ICRs is the best characterized mechanism by which imprinted gene expression is maintained in somatic cells. However, the relationship between ICR methylation and gene expression is not well understood for all imprinted genes. *Zfp57*^-/-^ ESCs display a specific DNA methylation defect at the 20 ICRs that are bound by ZFP57 in mouse pluripotent cells, and represent an ideal model to study the role of methylation in the control of imprinted gene expression during differentiation^24^. By employing an in vitro system of neural differentiation of hybrid ESCs, this study highlights the importance of ZFP57-dependent methylation in genomic imprinting, but also illustrates that imprinted gene control is more complex than what previously thought.

An unexpected finding of this study was that although the *Zfp57*^-/-^ cells were able to leave the pluripotency state they only partially differentiated into NPCs if compared to their derivative wild-type cells, under the conditions used in this study. We cannot exclude that the deregulated expression of some imprinted genes is secondary to the differentiation differences observed between the wild-type and mutant strains. Nevertheless, the general loss of allelic expression and the coordinated expression changes of the genes located in the same domain suggest that most of the imprinting loss is directly caused by lack of ZFP57-dependent ICR methylation.

Despite the presence of differential methylation at ICRs, allele-specific expression was well established at only a few genes in the undifferentiated ESCs. Some genes acquired imprinted expression after differentiation, others increased their allelic bias in NPCs. For several other genes, imprinting could be assessed only in NPCs, because their expression in ESCs was too low. Nevertheless, all the imprinted genes of wild-type NPCs, overall belonging to 15 of the 20 known imprinted gene clusters, lacked or reduced their gamete of origin-specific expression in *Zfp57*-mutant NPCs. This demonstrates that *Zfp57*-dependent methylation is generally required for both acquisition and maintenance of imprinted gene expression in somatic cells. Interestingly, some genes, such as *Nespas*, *Peg3*, *Plagl1*, *Meg3*, *Rian* and *Mirg*, partially retained their allelic bias in the *Zfp57*-mutant day 0-cells, but completely lost it in the *Zfp57*-mutant day 12-cells. Since both the *Zfp57*-mutant day 0 and *Zfp57*-mutant day 12-cells completely lack ICR methylation, this suggests that *Zfp57*-dependent methylation is the major determinant of imprinted gene expression in differentiated cells, but additional epigenetic mechanisms contribute to imprinting maintenance in pluripotent cells.

Lack of *Zfp57* leads to a more limited ICR methylation loss in mouse embryos than in cultured cells^17^. This is likely because the ESCs and the derived early differentiating cells undergo many more cell divisions than the equivalent cells in the early embryo, and there can thus be accumulation of small losses each cell cycle, eventually leading to fixation of the unmethylated state. So, an advantage of our ESC system is that it allows to score regions at which ZFP57 contributes only partly to the maintenance of methylation. The consequence of *Zfp57*-knockout on imprinted gene expression was investigated in mouse embryos in a very recent study^47^. The data of this study are consistent with ours, but are limited by an incomplete loss of ICR methylation of *Zfp57*-null embryos. Differently from our neural cell-based system, this in vivo study analyzed whole E13.5 embryos, which did therefore not allow detection of allelic expression of several tissue-specific imprinted genes. It is important to note, however, that because the *Zfp57* gene was inactivated prior to differentiation, none of these studies addresses the possible role of this gene in differentiated cells. In humans, *ZFP57* inactivation results in a less severe phenotype than in mice, possibly because *ZFP57* is not expressed in human oocytes and is activated only post-implantation, and another zinc-finger protein gene, *ZNF445*, appears to play a major role instead^18,25^.

Among the imprinted genes that were deregulated in the *Zfp57*-mutant NPLCs, some displayed up-regulation of the silenced allele, others down-regulation of the expressed allele, with respect to the wild-type cells. This behavior is consistent with the relationship between DMR methylation and allele-specific expression of each imprinted gene. In general, the gene *in cis* with DMR methylation acquired the expression of the other parental allele, in mutant cells. Thus, maternally expressed genes were repressed and paternally expressed genes activated in the domains with maternal ICR methylation. Conversely, maternally expressed genes were activated and paternally expressed genes repressed in the domains with paternal ICR methylation.

Intriguingly, several imprinted genes that were expected to be activated twofold—because now transcribed from both the parental alleles—did not significantly change their global expression level in *Zfp57*-mutant cells (Table 2). This suggests that compensatory mechanisms exist for many imprinted genes that finely control their RNA level. Most of these genes, interestingly, are expressed at a lower level in wild-type NPCs. Since their overall expression levels were not affected by expressing two alleles versus one, it may also be speculated that these genes are not the ones that have driven the evolution of imprinted gene expression. In the context of possible dosage compensation mechanisms, it is interesting to note that multiple regulatory links exist between different imprinted domains^48^. For instance, the maternally expressed miRNAs (miR-379/410) encoded by the *Dlk1-Meg3* imprinting domain antagonize paternally expressed transcripts from other imprinted domains, including *Plagl1*, *Peg3*, *Igf2* and *Mkrn3*^49,50^. The unexpected reduction in *Plagl1* and *Mkrn3* RNA could be explained by up-regulation of these miRNAs in the *Zfp57*-mutant day 12-cells (Supplementary Fig. 6a–c). This could also be responsible of the indirect down-regulation of several neuronal non-imprinted miRNA-target genes in the *Zfp57*^-/-^ NPLCs (Supplementary Fig. 3d).

*Zfp57-*mutant cells only partially differentiated towards neural precursors, suggesting that genes controlled by ZFP57 are necessary for neural differentiation of ESCs under the conditions used in this study. This finding contrasts with the absence of major problems of neurogenesis reported in *Zfp57* mutant embryos^17^. However, the *Zfp57*^-/-^ ESCs show more methylation defects at the ZFP57-binding sites and are therefore expected to have more gene expression changes and severe phenotype than the mutant embryos^24^. Consistent with this hypothesis, the *Dnmt3l* knockout that prevents establishment of methylation at all maternally imprinted DMRs is associated with exencephaly and other neural tube defects in the mouse^51^. A possible explanation of the partial differentiation capacity of the *Zfp57*^-/-^ cells toward the neural fate is the deregulation of imprinted genes that have a role in the control of neural cell proliferation, differentiation, apoptosis and migration, such as *Cdkn1c, Dlk1, Igf2, Peg3* and *Plagl1*^52–56^. Alternatively, the expression of non-imprinted genes involved in neurogenesis that are located close to the ZFP57 binding sites may be altered.

In conclusion, this study provides an extensive analysis of the relationship between DMR methylation and imprinted gene expression, and suggests that a number of ZFP57-target genes have a role in neural differentiation of mouse ESCs in vitro.

## Materials and methods

### Cell lines and culture conditions

The wild-type hybrid ESC line JB1, which is (JF1 × C57BL/6) F1, and the JB1-derived *Zfp57*^*-/-*^ ESC line were described previously^24,57^. ESCs were cultured under serum-free conditions on gelatinized tissue culture dishes and maintained in ESGRO Complete Plus Serum-free Clonal Grade 1i Medium (Merck-Millipore) in the presence of the Gsk3 inhibitor CHIR99021 (at 3 μM). The wild-type inbred 129 ESC line E14 and its derived *Zfp57*^-/-^ ESC line were cultured under standard feeder-free conditions as previously described^24^. The wild-type and *Zfp57*^-/-^ JB1 and E14 ESCs were differentiated toward NPCs following the protocol described by Gaspard and collaborators (Fig. 1a)^58^. In vitro corticogenesis of ESCs was started with plating at the optimal density of 7.500 ES cells/cm^2^ and culturing in chemically defined default medium (DDM). From day 2 to 10 of culture, a Sonic hedgehog inhibitor (cyclopamine) was added at 1 μM concentration. From day 10 to 12, the medium was replaced with DDM only. The cells were cultured at 37 °C under an atmosphere of 5% CO_2_.

### DNA methylation analysis

Genomic DNA from cultured cells was isolated using the Wizard Genomic DNA Purification Kit (Promega) following the manufacturer’s protocols. ~ 1 μg genomic DNA was subjected to bisulfite conversion using EpiTect Bisulfite Kit (Qiagen). The bisulfite sequencing was obtained by three methods: sequencing of individual clones, pyrosequencing and direct sequencing of the bisulfite-treated and PCR-amplified genomic DNA. PCR amplification was obtained by AmpliTaq DNA polymerase Kit (Thermo Fisher Scientific). For the *Plagl1*: TSS-DMR, *Peg13*: TSS-DMR and *Igf2r*: TSS-DMR, PCR products were cloned into the TOPO TA cloning vector (Invitrogen) and individual clones were isolated and sequenced (Eurofins Genomics). For the *H19/Igf2*:IG-DMR, *Mest*:TSS-DMR and *Rasgrf1*:TSS-DMR, DNA methylation was assessed by Sanger sequencing (Eurofins Genomics) of the bisulfite-treated and PCR-amplified genomic DNA (AmpliTaq DNA polymerase Kit, Thermo Fisher Scientific). For the *Kcnq1ot1*:TSS-DMR, *Meg3*/*Dlk1*:IG-DMR, *Peg3*:TSS-DMR, and *Snrpn*:TSS-DMR DNA methylation was analysed by pyrosequencing using PyroMark Q48 Autoprep sequencing machine (Qiagen). For the *H19/Igf2*:IG-DMR, *Mest*:TSS-DMR and *Rasgrf1*:TSS-DMR, DNA methylation was assessed after direct sequencing of the bisulfite-treated and PCR-amplified genomic DNA. All the primers are listed in Supplementary Table 6.

### RNA analysis

RNA was isolated using TRI reagent (Sigma-Aldrich) according to the manufacturer’s instructions. Approximately 900 ng of total RNA was retro-transcribed to cDNA by using QuantiTect Reverse Transcription Kit (Qiagen) according to the manufacturer protocol. For locus-specific gene expression analysis, cDNA was amplified by real-time PCR using SYBR Green PCR Master Mix (Bio-Rad) on a CFX Connect Real-Time PCR Detection System. For locus-specific allelic expression analysis, target genes were amplified, purified using PCR purification Kit (Qiagen), and sequenced using Sanger sequencing (Eurofins Genomics). PCR and RT-qPCR primers are reported in Supplementary Table 6.

For RNA-seq, ~ 1 μg of high quality RNA (260/280 > 1.80) samples in biological duplicates were subjected to library preparation using TruSeq Stranded Total RNA Ribo-Zero Gold Kit and sequencing with 2 × 125 bp paired end (PE) mode on Illumina HiSeq 2500 System (Institute of Applied Genomics: IGA, Italy).

### Bioinformatic analyses

The reads obtained from RNA-sequencing were assessed for quality using FastQC tool and trimmed for adapter sequences using Trimmomatic v.0.36 (in PE mode, MINLEN:36, HEADCROP if required and other parameters as default)^59^. Only good quality reads were aligned to the mouse reference genome (gencode vM20) using STAR v2.5.4a (-outFilterMultimapNmax 1, -outFilterMismatchNmax 3 and other parameters set as default)^60^. Reads were assigned to genes (annotation gencode vM20) using the featureCounts function (-p option) from Subread package v.1.6.0^61^. In the annotation file, two transcripts Gm49394 and Mir483, which overlapped *Igf2* coordinates and present in the gencode vM20 gtf annotation file, were removed. We arbitrarily set a cutoff of > 8 reads (wild-type + mutant samples) to remove the genes with very low expression level. We used DESeq2 v.1.24.0^29^ for normalization and differential expression between wildtype-NPCs and *Zfp57*^*-*/-^ NPCs. Genes with adjusted p-value < 0.05 were considered statistically significant and were used for downstream analysis. To determine the differentiation potential of our NPCs we compared our RNA-Seq data with publicly available RNA-Seq data of ESCs and NPCs^26^. The raw data were processed as before and the read count matrix was normalized by the “upper quartile” method. Subsequently, we adjusted the expression of all genes by dividing by beta-actin expression of the respective samples. After adjustment, we computed the Z-scores for each sample and plotted for a set of lineage-specific markers of pluripotency, neural/cortex, endoderm and mesoderm. The functional enrichment analysis of deregulated genes was performed using gprofiler2 *R package*^62^. Biological process terms (GO:BP) from gprofiler2 were considered significantly enriched if adjusted p-value < 0.05 and only top 10–15 candidates were depicted on a barplot. The allele-specific RNA-Seq analysis was performed by utilizing the SNPs present between B6 and JF1 genome. SNPs were derived from Takada and collaborators^63^ and converted to mm10 using CrossMap v-0.2.5^64^. These SNPs were overlapped with the JF1 SNPs downloaded from the Sanger Institute website (ftp://ftp-mouse.sanger.ac.uk/REL-1807-SNPs_Indels/mgp.v6.merged.norm.snp.indels.sfiltered.vcf.gz), which revealed ~ 11 millions SNP positions. These SNPs were used for creating N-masked genomes from mm10 reference genome using custom Python script by incorporating ‘Ns’ at the SNP positions. The alignment to N-masked genome was performed using STAR v2.5.4a with specific parameters (-alignEndsTypeEndToEnd, -outSAMattributes All, -outFilterMultimapNmax 1 -outFilterMismatchNmax 3 and other parameters as default). The aligned reads were assigned to maternal (JF1) or paternal (B6) allele using SNPSplit v0.3.2^30^, accompanied with a file containing SNPs information. For visualization purposes, bigwig files were generated and uploaded to UCSC genome browser (mouse genome version GRCm38/mm10). The allele-specific reads were assigned to the genes using featureCounts^61^ and imported to DESeq2 for further normalization. As both parental alleles (viz. B6 and JF1) were originated from the same sample, the allele-specific counts were normalised by size factor obtained from bulk RNA-Seq using DESeq2. We filtered out the genes with < 10 normalized counts (sum of mat and pat alleles) in both wild-type replicates. We confined our analysis only to imprinted genes, the list of which was collected from geneimprint database (www.geneimprint.com) and literature search^26^. The allele-specificity was estimated from the ratio of maternal allele normalized counts to the sum of maternal and paternal allele normalized counts (JF1/B6 + JF1) and only the genes with allelic ratio ≤ 0.33 or ≥ 0.67 were prioritized as monoallelic and plotted using ggplot2 *R package*. RNA-Seq raw and processed data is available in GEO (GSE164669).

ChIP-Seq data for ZFP57 and KAP1 were generated previously^20^ and available in GEO repository (GSE74757). The raw reads were downloaded and aligned to mm10 using bowtie v1.2.1.1 in single-end mode. Only the uniquely mapped reads were obtained (bowtie option -m 1) and PCR duplicates were removed using custom shell script. Peak were called by using macs14 (MACS 1.4.2)^65^, which revealed 544 ZFP57 and 6986 KAP1 peaks (Supplementary Table 7). To increase the confidence over identified peaks, both ZFP57 and KAP1 peaks were intersected using bedtools, which revealed 457 overlapping peaks. The distance of deregulated genes in the *Zfp57*^*-*/-^ day 12 cells from the ZFP57/KAP1 peaks was calculated using bedtools (-closest function) and plotted using ggplot2 *R package*. The genes overlapping with ZFP57/KAP1 peaks were set at distance 0.

### Statistical analysis

Statistical significance for the relative expression of the lineage-specific markers and imprinted genes was conducted by unpaired Student’s t-test. Enrichment of differentially expressed imprinted genes over all differentially expressed genes was assessed using the Hypergeometric test. The difference between the two empirical cumulative functions of the distances between genes and ZFP57/KAP1 peaks was evaluated using the two-sample Kolmogorov–Smirnov test. The allele-specific expression of genes in wild-type cells was assessed using a Proportion test and multiplicity correction was performed using the “Benjamini-Hochberg” method.

### Immunofluorescence

For Nestin and Pou5f1 ( also known as Oct3-4) immunofluorescence, the cells were fixed in 4% PFA and permeabilized with 0.1% Triton X-100/PBS. Cells were, then, blocked with 10% normal goat serum (NGS)/ 1% BSA(bovine serum albumin)/ PBS. Later, the cells were incubated with the primary antibodies (i.e., anti-Nestin, anti-Pou5f1, conjugated respectively with Alexa Fluor 594, sc-33677 AF594 and Alexa Fluor 488, sc-5279 AF488). After 3 washes with 0.1% Triton X-100/PBS cells were incubated with DAPI for 5 min. Images were captured with a fluorescence microscope (DMI6000B; Leica) run with the LAS AF 2.6 software (Leica).

## Supplementary Information

Supplementary Figures.

Supplementary Tables.

## References

[1] Plasschaert RN, Bartolomei MS (2014). Genomic imprinting in development, growth, behavior and stem cells. Development.

[2] Morison IM, Ramsay JP, Spencer HG (2005). A census of mammalian imprinting. Trends Genet..

[3] Skaar DA (2012). The human imprintome: Regulatory mechanisms, methods of ascertainment, and roles in disease susceptibility. ILAR J..

[4] Blake A (2010). MouseBook: An integrated portal of mouse resources. Nucleic Acids Res..

[5] Andergassen, D. *et al.* Mapping the mouse allelome reveals tissue-specific regulation of allelic expression. *Elife* **6**. 10.7554/eLife.25125 (2017). 10.7554/eLife.25125 PMC5555720 28806168

[6] Soellner L (2017). Recent advances in imprinting disorders. Clin. Genet..

[7] MacDonald WA, Mann MR (2014). Epigenetic regulation of genomic imprinting from germ line to preimplantation. Mol. Reprod. Dev..

[8] Monk D, Mackay DJG, Eggermann T, Maher ER, Riccio A (2019). Genomic imprinting disorders: Lessons on how genome, epigenome and environment interact. Nat. Rev. Genet..

[9] Li E, Beard C, Jaenisch R (1993). Role for DNA methylation in genomic imprinting. Nature.

[10] Kaneda M (2004). Essential role for de novo DNA methyltransferase Dnmt3a in paternal and maternal imprinting. Nature.

[11] Lewis A (2004). Imprinting on distal chromosome 7 in the placenta involves repressive histone methylation independent of DNA methylation. Nat. Genet..

[12] Caspary T, Cleary MA, Baker CC, Guan XJ, Tilghman SM (1998). Multiple mechanisms regulate imprinting of the mouse distal chromosome 7 gene cluster. Mol. Cell Biol..

[13] Tanaka M (1999). Parental origin-specific expression of Mash2 is established at the time of implantation with its imprinting mechanism highly resistant to genome-wide demethylation. Mech. Dev..

[14] El Kharroubi A, Piras G, Stewart CL (2001). DNA demethylation reactivates a subset of imprinted genes in uniparental mouse embryonic fibroblasts. J. Biol. Chem.

[15] Inoue A, Jiang L, Lu F, Suzuki T, Zhang Y (2017). Maternal H3K27me3 controls DNA methylation-independent imprinting. Nature.

[16] Chen Z, Zhang Y (2020). Maternal H3K27me3-dependent autosomal and X chromosome imprinting. Nat. Rev. Genet..

[17] Li X (2008). A maternal-zygotic effect gene, Zfp57, maintains both maternal and paternal imprints. Dev. Cell.

[18] Mackay DJ (2008). Hypomethylation of multiple imprinted loci in individuals with transient neonatal diabetes is associated with mutations in ZFP57. Nat. Genet..

[19] Quenneville S (2011). In embryonic stem cells, ZFP57/KAP1 recognize a methylated hexanucleotide to affect chromatin and DNA methylation of imprinting control regions. Mol. Cell.

[20] Anvar Z (2016). ZFP57 recognizes multiple and closely spaced sequence motif variants to maintain repressive epigenetic marks in mouse embryonic stem cells. Nucleic Acids Res..

[21] Messerschmidt DM (2012). Trim28 is required for epigenetic stability during mouse oocyte to embryo transition. Science.

[22] Strogantsev R (2015). Allele-specific binding of ZFP57 in the epigenetic regulation of imprinted and non-imprinted monoallelic expression. Genome Biol..

[23] Shi H (2019). ZFP57 regulation of transposable elements and gene expression within and beyond imprinted domains. Epigenet. Chromatin.

[24] Riso V (2016). ZFP57 maintains the parent-of-origin-specific expression of the imprinted genes and differentially affects non-imprinted targets in mouse embryonic stem cells. Nucleic Acids Res..

[25] Takahashi N (2019). ZNF445 is a primary regulator of genomic imprinting. Genes Dev..

[26] Bouschet T (2017). In vitro corticogenesis from embryonic stem cells recapitulates the in vivo epigenetic control of imprinted gene expression. Cereb. Cortex.

[27] Gaspard N (2009). Generation of cortical neurons from mouse embryonic stem cells. Nat. Protoc..

[28] Akagi T (2005). Identification of Zfp-57 as a downstream molecule of STAT3 and Oct-3/4 in embryonic stem cells. Biochem. Biophys. Res. Commun..

[29] Love MI, Huber W, Anders S (2014). Moderated estimation of fold change and dispersion for RNA-seq data with DESeq2. Genome Biol..

[30] Krueger, F. & Andrews, S. R. SNPsplit: Allele-specific splitting of alignments between genomes with known SNP genotypes. *F1000Res* **5**, 1479. 10.12688/f1000research.9037.2 (2016). 10.12688/f1000research.9037.1 PMC4934512 27429743

[31] Duffie R (2014). The Gpr1/Zdbf2 locus provides new paradigms for transient and dynamic genomic imprinting in mammals. Genes Dev..

[32] DeVeale B, van der Kooy D, Babak T (2012). Critical evaluation of imprinted gene expression by RNA-Seq: A new perspective. PLoS Genet..

[33] Kagitani F (1997). Peg5/Neuronatin is an imprinted gene located on sub-distal chromosome 2 in the mouse. Nucleic Acids Res..

[34] Schulz R (2009). Transcript- and tissue-specific imprinting of a tumour suppressor gene. Hum. Mol. Genet..

[35] Tibbit CJ (2015). Antisense activity across the Nesp promoter is required for Nespas-mediated silencing in the imprinted Gnas cluster. Noncoding RNA.

[36] Ono R (2003). Identification of a large novel imprinted gene cluster on mouse proximal chromosome 6. Genome Res..

[37] Babak T (2008). Global survey of genomic imprinting by transcriptome sequencing. Curr. Biol..

[38] MacIsaac JL, Bogutz AB, Morrissy AS, Lefebvre L (2012). Tissue-specific alternative polyadenylation at the imprinted gene Mest regulates allelic usage at Copg2. Nucleic Acids Res..

[39] Brideau CM, Eilertson KE, Hagarman JA, Bustamante CD, Soloway PD (2010). Successful computational prediction of novel imprinted genes from epigenomic features. Mol. Cell Biol..

[40] Ye A, He H, Kim J (2014). Paternally expressed Peg3 controls maternally expressed Zim1 as a trans factor. PLoS ONE.

[41] Ideraabdullah FY, Bartolomei MS (2011). ZFP57: KAPturing DNA methylation at imprinted loci. Mol. Cell.

[42] Okae H (2012). Re-investigation and RNA sequencing-based identification of genes with placenta-specific imprinted expression. Hum. Mol. Genet..

[43] Iglesias-Platas I (2013). Imprinting at the PLAGL1 domain is contained within a 70-kb CTCF/cohesin-mediated non-allelic chromatin loop. Nucleic Acids Res..

[44] Hikichi T, Kohda T, Kaneko-Ishino T, Ishino F (2003). Imprinting regulation of the murine Meg1/Grb10 and human GRB10 genes; roles of brain-specific promoters and mouse-specific CTCF-binding sites. Nucleic Acids Res..

[45] Sanli I (2018). Meg3 non-coding RNA expression controls imprinting by preventing transcriptional upregulation in cis. Cell Rep..

[46] Latos PA (2012). Airn transcriptional overlap, but not its lncRNA products, induces imprinted Igf2r silencing. Science.

[47] Jiang, W. *et al.* ZFP57 dictates allelic expression switch of target imprinted genes. *Proc. Natl. Acad. Sci. USA* **118**. 10.1073/pnas.2005377118 (2021). 10.1073/pnas.2005377118 PMC7865185 33500348

[48] Varrault A (2017). Identification of Plagl1/Zac1 binding sites and target genes establishes its role in the regulation of extracellular matrix genes and the imprinted gene network. Nucleic Acids Res..

[49] Whipple, A. J. *et al.* Imprinted maternally expressed microRNAs antagonize paternally driven gene programs in neurons. *Mol. Cell* **78**, 85–95 e88. 10.1016/j.molcel.2020.01.020 (2020). 10.1016/j.molcel.2020.01.020 PMC7176019 32032531

[50] Ghousein A, Feil R (2020). Imprinted small RNAs unraveled: Maternal microRNAs antagonize a paternal-genome-driven gene expression network. Mol. Cell.

[51] Bourc'his D, Xu GL, Lin CS, Bollman B, Bestor TH (2001). Dnmt3L and the establishment of maternal genomic imprints. Science.

[52] Ferron SR (2011). Postnatal loss of Dlk1 imprinting in stem cells and niche astrocytes regulates neurogenesis. Nature.

[53] Adnani L (2015). Zac1 regulates the differentiation and migration of neocortical neurons via Pac1. J. Neurosci..

[54] Johnson MD, Wu X, Aithmitti N, Morrison RS (2002). Peg3/Pw1 is a mediator between p53 and Bax in DNA damage-induced neuronal death. J. Biol. Chem..

[55] Mairet-Coello G (2012). p57(KIP2) regulates radial glia and intermediate precursor cell cycle dynamics and lower layer neurogenesis in developing cerebral cortex. Development.

[56] Lehtinen MK (2011). The cerebrospinal fluid provides a proliferative niche for neural progenitor cells. Neuron.

[57] Kota SK (2014). ICR noncoding RNA expression controls imprinting and DNA replication at the Dlk1-Dio3 domain. Dev. Cell.

[58] Gaspard N (2008). An intrinsic mechanism of corticogenesis from embryonic stem cells. Nature.

[59] Bolger AM, Lohse M, Usadel B (2014). Trimmomatic: A flexible trimmer for Illumina sequence data. Bioinformatics.

[60] Dobin A (2013). STAR: Ultrafast universal RNA-seq aligner. Bioinformatics.

[61] Liao Y, Smyth GK, Shi W (2014). featureCounts: An efficient general purpose program for assigning sequence reads to genomic features. Bioinformatics.

[62] Raudvere U (2019). g:Profiler: A web server for functional enrichment analysis and conversions of gene lists (2019 update). Nucleic Acids Res..

[63] Takada T (2013). The ancestor of extant Japanese fancy mice contributed to the mosaic genomes of classical inbred strains. Genome Res..

[64] Zhao H (2014). CrossMap: A versatile tool for coordinate conversion between genome assemblies. Bioinformatics.

[65] Zhang Y (2008). Model-based analysis of ChIP-Seq (MACS). Genome Biol..

