# Supplementary material for: Zfp57 inactivation illustrates the role of ICR methylation in imprinted gene expression during neural differentiation of mouse ESCs
Source: Sci Rep. 2021 Jul 5;11:13802. doi: 10.1038/s41598-021-93297-3 (PMC8257706; doi:10.1038/s41598-021-93297-3)
Supplement: Supplementary file 1 — Supplementary Figures. [file 41598_2021_93297_MOESM1_ESM.pdf]

Zfp57 inactivation illustrates the role of ICR methylation in imprinted gene expression during neural differentiation of mouse ESCs

Basilia Acurzio[1,2#], Ankit Verma[1,2#], Alessia Polito[2,3¶], Carlo Giaccari[1,2], Francesco Cecere[1,2], Salvatore Fioriniello[2], Floriana Della Ragione[2], Annalisa Fico[2], Flavia Cerrato[1], Claudia Angelini[4], Robert Feil[5,6], Andrea Riccio[1,2*]

[1] Department of Environmental Biological and Pharmaceutical Sciences and Technologies (DiSTABiF), Università degli Studi della Campania "Luigi Vanvitelli", 81100 Caserta, Italy.
[2] Institute of Genetics and Biophysics (IGB) "Adriano Buzzati-Traverso", Consiglio Nazionale delle Ricerche (CNR), 80131 Naples, Italy.
[3] Department of Biology, Università degli Studi di Napoli "Federico II", 80126 Napoli, Italy.
[4] Istituto per le Applicazioni del Calcolo "Mauro Picone" (IAC), CNR, 80131 Naples, Italy.
[5] Institute of Molecular Genetics of Montpellier (IGMM), Centre National de Recherche Scientifique (CNRS), 34293 Montpellier, France.
[6] University of Montpellier, 34090 Montpellier, France.

* Corresponding author: andrea.riccio@unicampania.it, tel +39 0823 274599
# These authors contributed equally.
¶Present address: Department of Neuroscience and Brain Technologies - Istituto Italiano di Tecnologia,16163 Genova, Italy.

SUPPLEMENTARY INFORMATION

Legends to supplementary Figures

Supplementary Figure 1. Immunofluorescence (IF) staining of Nestin and Pou5f1(Oct3-4) in wild-type and *Zfp57*-/- at d12 JB1 cells of cyclopamine-mediated differentiation. Representative images of undifferentiated and mESC-derived neural precursor wild-type and Zfp57-/- JB1 cells examined by immuno-staining using anti-Nestin and anti-Pou5f1 antibodies. Nuclei were stained with DAPI. Merge: Nestin/ Oct3-4/DAPI signals. Scale bars, 100 µm

Supplementary Figure 2. Differentiation and imprinted genes expression analysis of wild-type and *Zfp57*$^{-/-}$ inbred cells. (**a**)Representative images of undifferentiated and mESC-derived neural precursor wild-type and Zfp57-/- cells examined by immuno-staining using anti-Nestin and anti-Pou5f1 antibodies. Nuclei were stained with DAPI. Merge: Nestin/ Oct3-4/DAPI signals. Scale bars, 100 µm. (**b,c**) Expression analysis of imprinted genes (b,c) in wild-type and *Zfp57*$^{-/-}$ E14 cells at day 0 and day 12 assayed by quantitative RT-qPCR. The histograms show the average gene expression levels of three independent experiments, after normalization against the level of *β-actin*. Error bars represent the SD.

Supplementary Figure 3. Analysis of DMR methylation at ICRs in wild-type and *Zfp57*$^{-/-}$ NPCs. (**a**). Allele-specific DNA methylation results of three DMRs (*Plagl1*, *Peg13* and *Igf2r)* obtained by bisulfite treatment followed by cloning and sequencing. Each row corresponds to a single template DNA molecule cloned; each circle corresponds to a CpG dinucleotide. Filled circles designate methylated cytosines; open circles, unmethylated cytosines. The SNP used to distinguish the parental alleles is reported. (**b, c**) DNA methylation analysis of the *Snrpn:*TSS-DMR, *Peg3:*TSS-DMR, *Kcnq1ot1*:TSS-DMR and *Meg3/Dlk1*:IG-DMR by bisulfite treatment and pyrosequencing in JB1(b) and E14 (c) cell lines (wild-type and Zfp57-/-) at day 12. Violin-plots represent the average methylation level of several CpG of the DMR expressed in %mCpG. 'n' represents the number of CpGs analysed. The box-plot inside the violin-plot indicates interquartile range (25%-75%) and median value. (**d**) DNA methylation profiling of the *Mest:*TSS-DMR, *Rasgrf1*:TSS-DMR and *H19/Igf2*:IG-DMR, by bisulfite treatment and Sanger sequencing. The arrows above the electropherograms indicate the position of the CpGs. After bisulfite conversion, methylated cytosines appear as C and non-methylated cytosines as T.

Supplementary Figure 4. Allele-specific expression analysis of imprinted genes in wild-type and *Zfp57*$^{-/-}$ NPCs. Transcribed regions encompassing SNPs were analysed by Sanger sequencing after RT-PCR. Red arrows indicate the position of SNPs used to distinguish paternal (P) and maternal (M) alleles are indicated by red arrows.

Supplementary Figure 5. Deregulated expression at the polycistronic *Meg3* RNA in *Zfp57*$^{-/-}$ NPCs. (**A**) Screen shot from the UCSC Genome Browser depicting location of transcripts, and the results of bulk (black) and allele-specific (pink for maternal, blue for paternal) RNA-seq analysis of wild-type and *Zfp57*$^{-/-}$ JB1 NPCs. (**B-D**) Deregulation of bulk RNA level of the miRNA 379/410 cluster (B), their target imprinted genes *Mkrn3*, *Plagl1* and *Peg3* (C) and their target synaptic transmission and activity markers (D), expressed as log-ratio between *Zfp57*$^{-/-}$ and wild-type NPCs.

Supplementary Tables

Supplementary Table 1. RNA-seq data retrieved from GEO
Supplementary Table 2. Differentially expressed genes in *Zfp57*$^{-/-}$ day 12-JB1 cells (n=4912)

Supplementary Table 3. Downregulated genes within 100 kb from the ZFP57 binding sites in *Zfp57*$^{-/-}$ JB1 day 12-cells.
Supplementary Table 4. Allele-specific gene expression analysis in wild-type and *Zfp57*$^{-/-}$ JB1 day 12-cells.
Supplementary Table 5. Allele-specific expression of imprinted genes in wild-type and *Zfp57*$^{-/-}$ JB1 day 0 and day 12-JB1 cells.
Supplementary Table 6. Sequence of PCR primers used in this study.
Supplementary Table 7. ZFP57 and KAP1 overlapping peaks in wild-type day 0-JB1 cells.

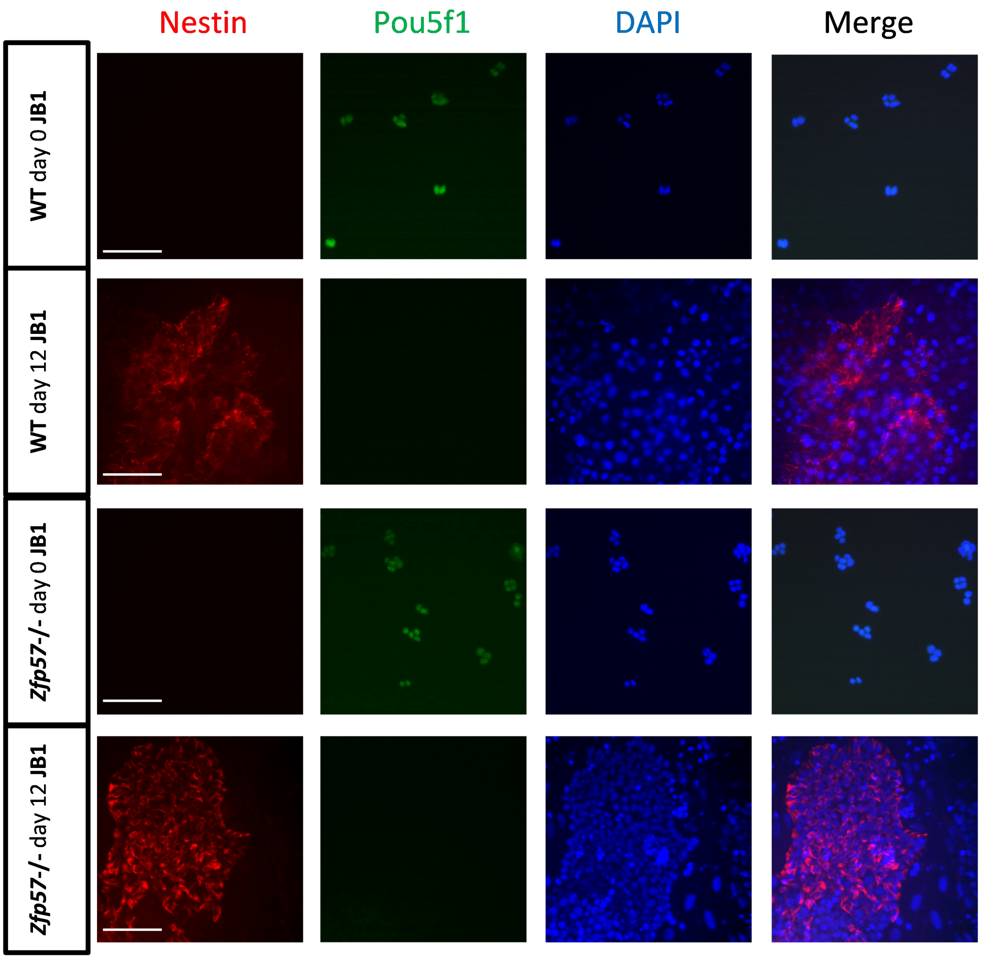

Supplementary Figure 1

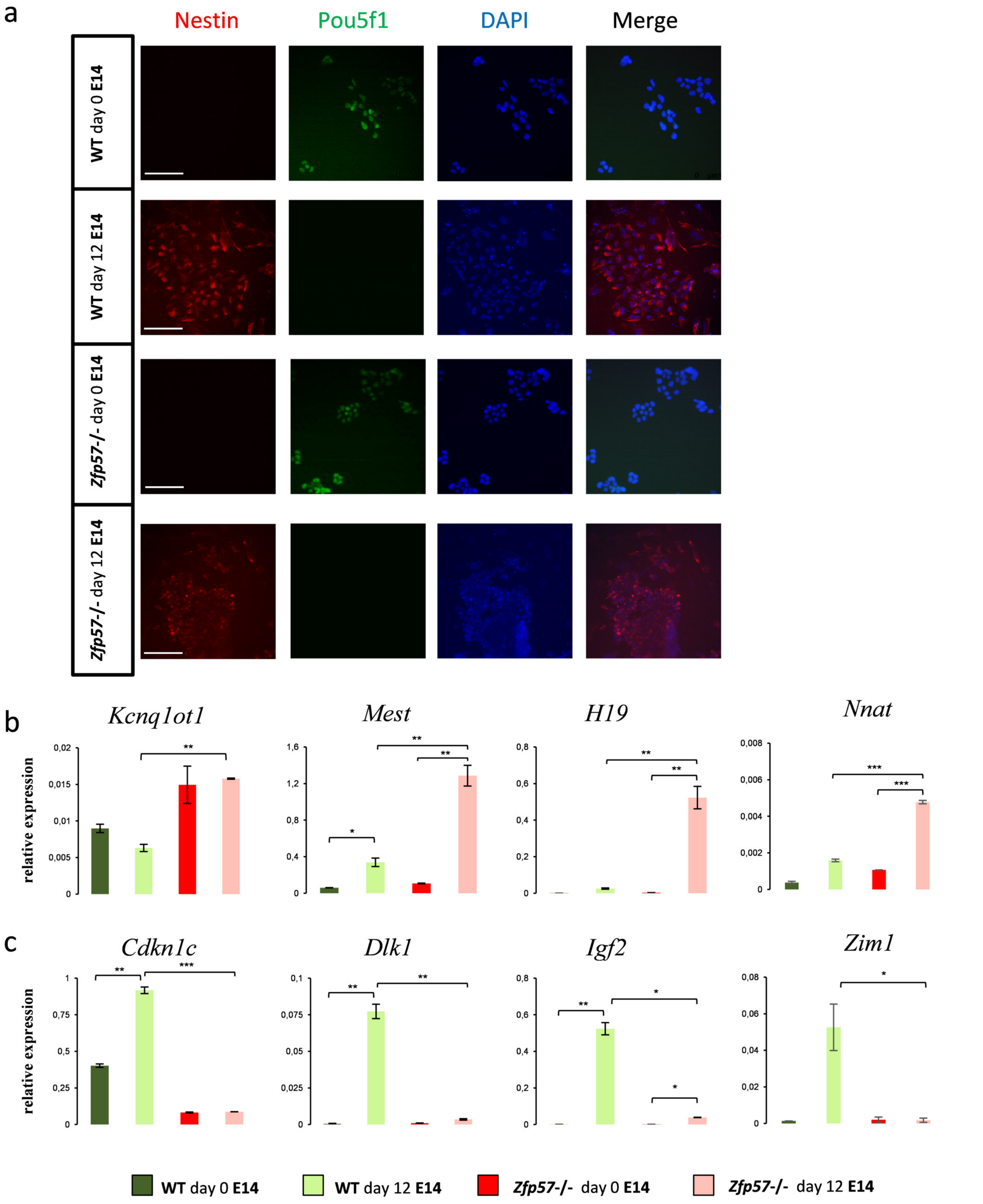

Supplementary Figure 2

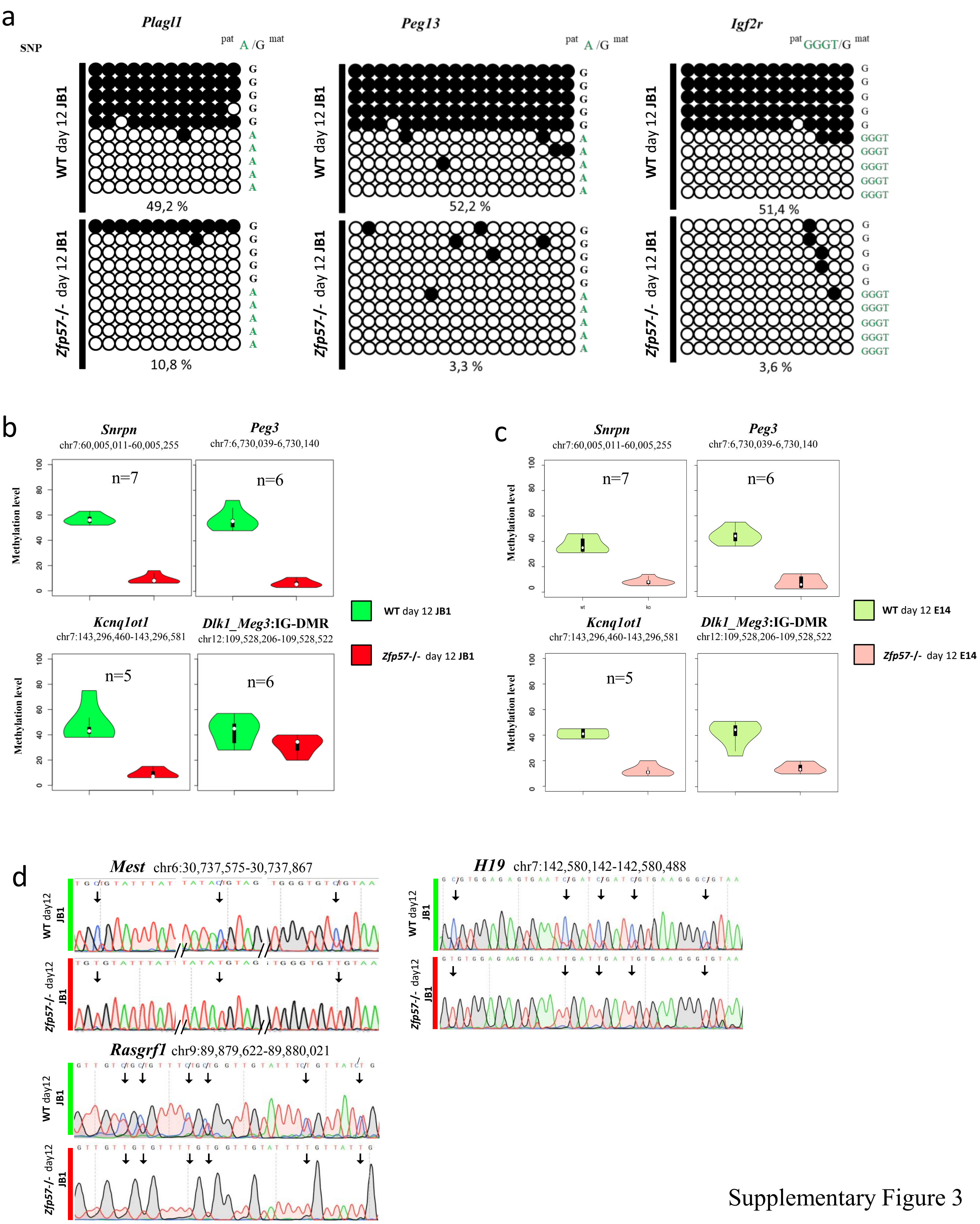

Supplementary Figure 3

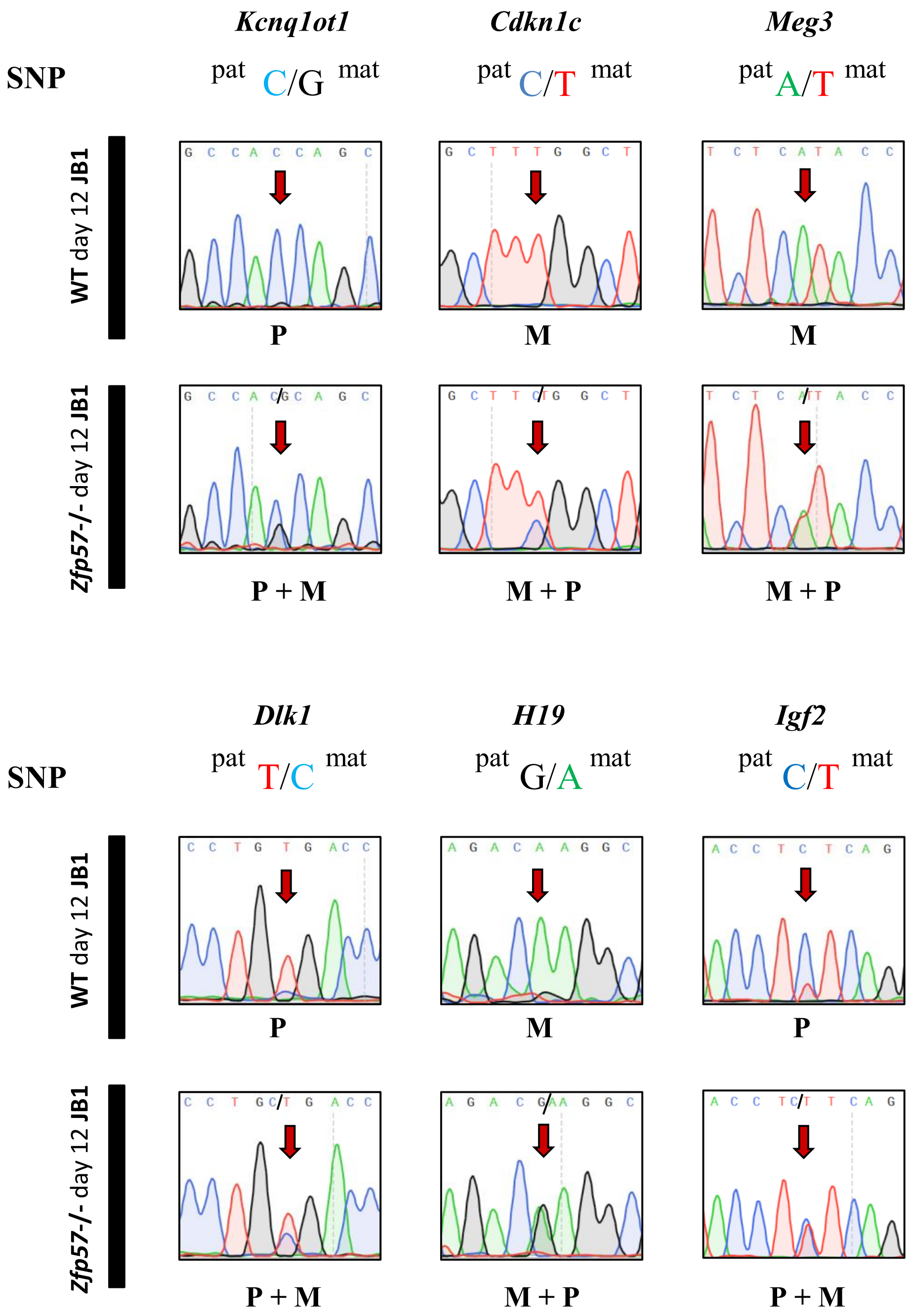

Supplementary Figure 4

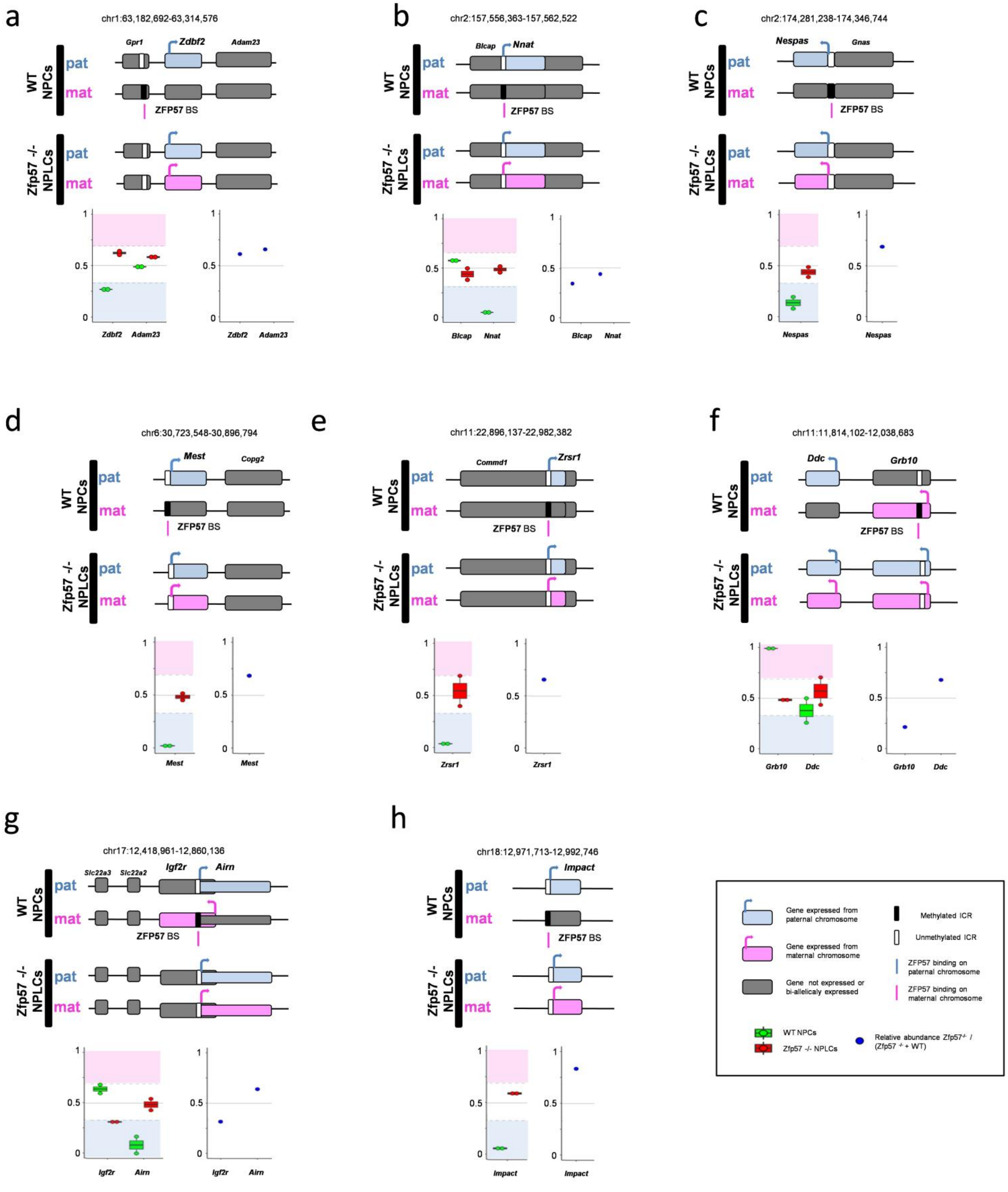

Supplementary Figure 5

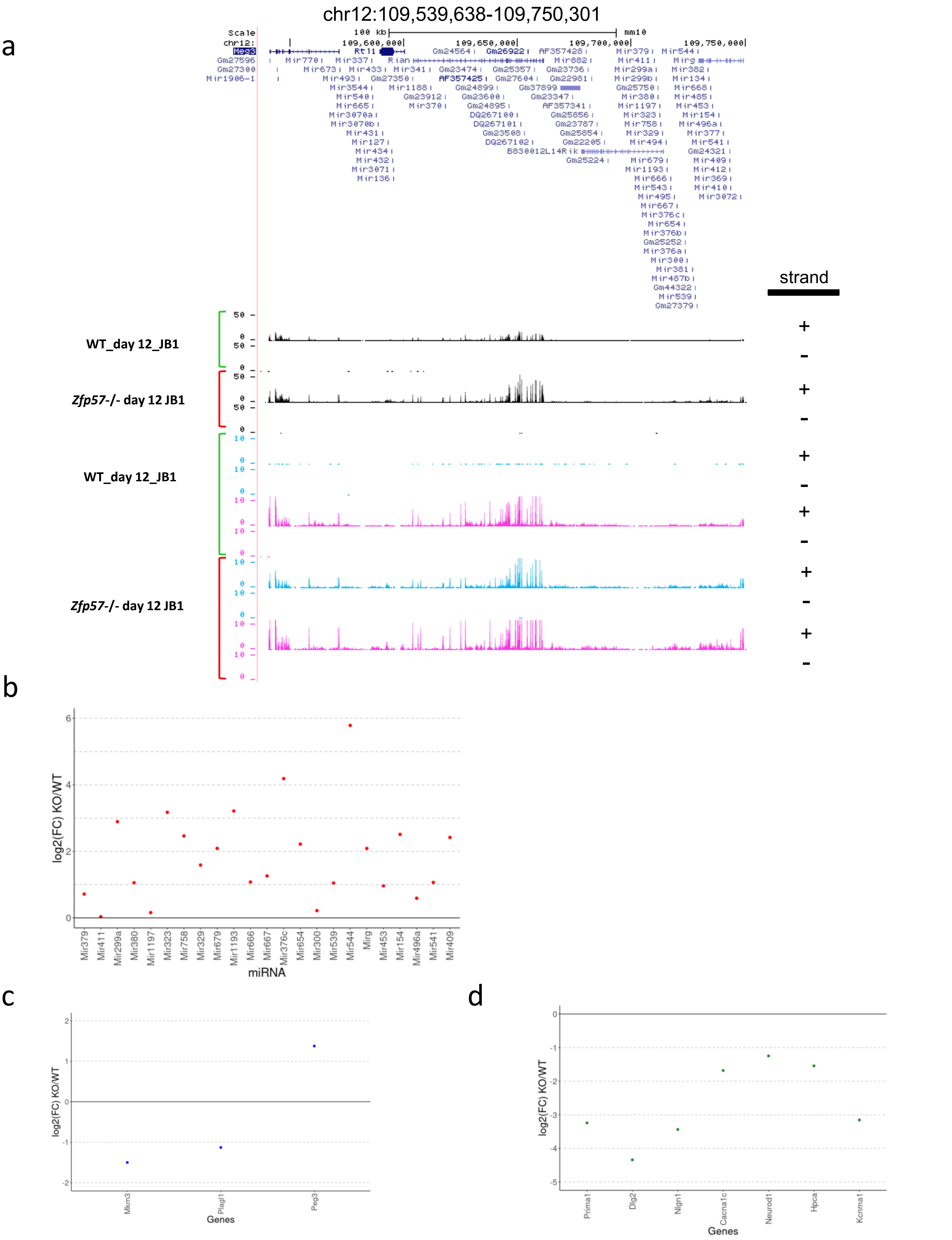

Supplementary Figure 6
